# Biosensor‐Enabled Multiplexed On‐Site Therapeutic Drug Monitoring of Antibiotics

**DOI:** 10.1002/adma.202104555

**Published:** 2021-09-21

**Authors:** H. Ceren Ates, Hasti Mohsenin, Christin Wenzel, Regina T. Glatz, Hanna J. Wagner, Richard Bruch, Nico Hoefflin, Sashko Spassov, Lea Streicher, Sara Lozano‐Zahonero, Bernd Flamm, Rainer Trittler, Martin J. Hug, Maja Köhn, Johannes Schmidt, Stefan Schumann, Gerald A. Urban, Wilfried Weber, Can Dincer

**Affiliations:** ^1^ FIT Freiburg Center for Interactive Materials and Bioinspired Technologies University of Freiburg Georges‐Koehler‐Allee 105 79110 Freiburg Germany; ^2^ Department of Microsystems Engineering (IMTEK) Laboratory for Sensors University of Freiburg Georges‐Koehler‐Allee 103 79110 Freiburg Germany; ^3^ Faculty of Biology and Signalling Research Centres BIOSS and CIBSS University of Freiburg Schaenzlestrasse 18 79104 Freiburg Germany; ^4^ Department of Anesthesiology and Critical Care Faculty of Medicine Medical Center – University of Freiburg Hugstetter Str. 55 79106 Freiburg Germany; ^5^ Department of Biosystems Science and Engineering ETH Zurich Mattenstrasse 26 Basel 4058 Switzerland; ^6^ Department of Pharmacy Medical Center – University of Freiburg Hugstetter Straße 55 79106 Freiburg Germany; ^7^ Freiburg Materials Research Center (FMF) University of Freiburg Stefan‐Meier‐Straße 21 79104 Freiburg Germany

**Keywords:** ß‐lactam antibiotics, exhaled breath condensate, multiplexing, noninvasive diagnostics, point‐of‐care testing

## Abstract

Personalized antibiotherapy ensures that the antibiotic concentration remains in the optimal therapeutic window to maximize efficacy, minimize side effects, and avoid the emergence of drug resistance due to insufficient dosing. However, such individualized schemes need frequent sampling to tailor the blood antibiotic concentrations. To optimally integrate therapeutic drug monitoring (TDM) into the clinical workflow, antibiotic levels can either be measured in blood using point‐of‐care testing (POCT), or can rely on noninvasive sampling. Here, a versatile biosensor with an antibody‐free assay for on‐site TDM is presented. The platform is evaluated with an animal study, where antibiotic concentrations are quantified in different matrices including whole blood, plasma, urine, saliva, and exhaled breath condensate (EBC). The clearance and the temporal evaluation of antibiotic levels in EBC and plasma are demonstrated. Influence of matrix effects on measured drug concentrations is determined by comparing the plasma levels with those in noninvasive samples. The system's potential for blood‐based POCT is further illustrated by tracking ß‑lactam concentrations in untreated blood samples. Finally, multiplexing capabilities are explored successfully for multianalyte/sample analysis. By enabling a rapid, low‐cost, sample‐independent, and multiplexed on‐site TDM, this system can shift the paradigm of “one‑size‐fits‐all” strategy.

## Graphical abstract

The first electrochemical biosensor, with a synthetic‐biology‐enabled antibody‐free assay, for temporal monitoring of piperacillin/tazobactam in exhaled breath condensate, along with a correlation study exploring the link between monitored drug levels, is presented. The validation of the findings with gold‐standard measurements demonstrates the suitability of the system as a low‐cost, rapid, and sample‐independent tool for multiplexed on‐site therapeutic drug monitoring.

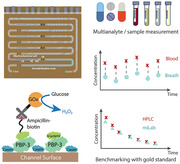

## 1 Introduction

We are at risk of losing the power of antibiotics, as the rate of antibiotic resistance is rising to dangerous levels. Newly developed agents are becoming ineffective much more rapidly than during the previous decades, while the celerity of our new inventions alarmingly falls behind. This bottleneck mandates the re‐evaluation of our battle strategy about how we utilize the existing antibiotics.

Therapeutic drug monitoring (TDM) is the clinical practice of measuring this drug concentration in blood or plasma, or in other biological fluids that can be linked to blood drug levels. Success of the antibiotherapy strongly depends on the ability to keep the antibiotic concentrations within therapeutic ranges tailored to respond the unique pharmacokinetics/pharmacodynamics (PK/PD) of the patient. In the current practice, however, this operational window is determined based on the data collected from animal models and healthy population. Based on these statistically accepted ranges, patients’ drug concentrations are then categorized as either subtherapeutic, therapeutic, or toxic. Therefore, physicians can only judge the presence or absence of a clinical response days later. In such a case, either a different type of antibiotic is utilized or additional agents are added to the therapy.^[^ ^1^ ^]^ In this regard, traditional TDM would be an extension of the “one‐size‐fits‐all” approach, potentially resulting in subtherapeutic conditions and in turn, antibiotic resistance.^[^ ^1^, ^2^, ^3^, ^4^, ^5^ ^]^ Given the variations in the antibiotic exposures across different patients, personalized antibiotherapy is considered as a promising remedy to maximize the antibiotic effectiveness. In such an individualized approach, the dynamics of the treatment process is to be tailored simultaneously, according to the requirements of each individual.^[^ ^1^ ^]^ This process necessitates a feedback control loop, bringing a much greater burden to the clinical laboratory because samples need to be analyzed more frequently to tailor both the PK/PD models and the therapeutic targets. Implementation of data‐driven methods, like Bayesian forecasting, to feedback the control loop can further support the dose optimization. Another challenge here is the paucity of valid assays for many drugs of interest to be monitored.

Therapeutic studies have been mostly established for blood‐based analysis, providing a relatively voluminous database compared to other bodily fluids. Nonetheless, this familiar approach is impractical considering the costs and resources associated with collecting, transporting, processing, and analyzing the blood for personalized TDM. Recent blood‐based studies are focusing on ways to alleviate these issues by decreasing the sample volume and eliminating the need for expensive equipment and expertise.^[^ ^6^, ^7^, ^8^, ^9^, ^10^, ^11^ ^]^ Alternatively, other matrices such as interstitial fluid,^[^ ^12^, ^13^, ^14^, ^15^, ^16^, ^17^, ^18^ ^]^ tears,^[^ ^19^ ^]^ saliva,^[^ ^20^, ^21^, ^22^ ^]^ or sweat^[^ ^22^, ^23^, ^24^, ^25^, ^26^, ^27^, ^28^, ^29^ ^]^ can be used to replace the invasive TDM. Herein, the common challenges stem from the complex transport mechanisms of antibiotics from the blood to the sampling site of interest, which make the interpretation of the measured concentrations unique for each medium. Breath could be a potential alternative to bypass the transportation‐related issues, as blood–breath transportation is relatively direct compared with the other noninvasive samples. In particular, transport resistances result in low analyte concentrations, necessitating highly sensitive detection methods, while secretion of the noninvasive samples further complicates the way the antibiotics interact with metabolic activities.^[^ ^30^ ^]^

Recently, a fluorescence spectrometry method coupled with copper nanocrystals has been introduced for the determination of vancomycin in exhaled breath condensate (EBC) samples after the administration of the antibiotic.^[^ ^31^ ^]^ Although the possibility of detecting vancomycin in EBC was demonstrated with a spike–recovery test, the temporal evaluation of vancomycin in EBC samples and correlation of measured values with plasma levels have not been investigated. In a similar study, UV–vis spectroscopy has been utilized for tobramycin detection from breath samples of healthy subjects inhaling antibiotics for a certain amount of time.^[^ ^32^ ^]^ However, also in this study, no investigation has been performed to correlate antibiotic levels in plasma and EBC. Some attempts have been made to quantify different types of antibiotics in EBC using chromatographic methods. Breath and EBC samples from patients given piperacillin/tazobactam or meropenem were measured using ultrahigh‐pressure liquid chromatography high‐resolution mass spectrometry.^[^ ^33^ ^]^ While the possibility of a correlation was examined, no link between EBC and plasma antibiotic levels could be found. To the best of our knowledge, the in‐depth study of time‐dependent correlation between antibiotic levels in EBC and plasma using biosensors remains unexplored.

Consequently, there is still no consensus in the community on how to interpret the measured concentrations and how to correlate them with blood‐based measurements. In clinical practice, this uncertainty is translated into the following questions: i) can we measure the antibiotic concentrations sensitive enough to perform PK/PD analysis? ii) how can we identify the effect of transport mechanisms on measured concentrations? and iii) can we quantify the instantaneous correlation between blood and noninvasive samples, preferably on the same platform?

**Figure 1:**
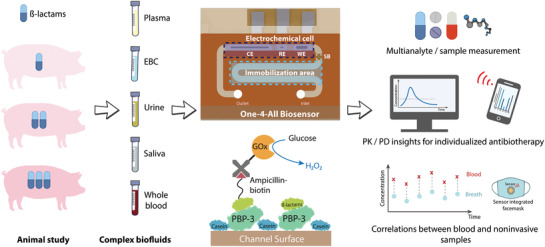
Utilization of proposed microfluidic biosensor (miLab) with the envisioned POC scenario. Both invasive and noninvasive samples collected from Landrace pigs given overdose, normal dose, and underdose piperacillin/tazobactam are analyzed using our electrochemical biosensor. The miLab chip consists of two consecutive zones separated by a hydrophobic stopping barrier. By separating the electrochemical detection zone from immobilization area, our platform can bypass the electrode fouling issue and operate with complex biofluids, like whole blood. A competitive and antibody‐free assay using penicillin‐binding proteins enables a rapid (less than 90 min) and highly sensitive (ng mL^−1^ range) detection of ß‐lactams. Combining with multiplexed microfluidics, our biosensor has the potential to be used in multianalyte/sample measurements as well as PK/PD and correlation studies for individualized drug therapy.

To answer these questions, we introduce a versatile, polymer‐based, disposable microfluidic sensor platform along with an antibody‐free and highly sensitive ß‐lactam assay (**Figure** **1**) and explore its capabilities with animal experiments conducted on Landrace pigs treated with three different (under‐, over‐, and normal) dosages of piperacillin/tazobactam. We successfully demonstrate, for the first time, the detection and temporal monitoring of piperacillin/tazobactam in EBC, along with a correlation study exploring the link between plasma and EBC drug levels. Hereby, we employ a biosensor with a high‐performance synthetic‐biology‐enableed assay enabling measurement of very low (ng mL^−1^ range) drug concentrations, which is not possible to achieve with conventional chromatography‐based methods^[^ ^34^ ^]^ (Table S3, Supporting Information). We benchmark our platform with high‐performance liquid chromatography (HPLC) measurements (gold standard) by comparing the plasma ß‐lactam concentrations of pigs given standard dosage.We further examine the impact of matrix effects by comparing plasma concentrations with those in EBC, saliva, and urine. We then survey the possibility of tracking concentration variations in untreated whole blood samples to explore the potential of our implemented biosensing system for point‐of‐care (POC) applications. Finally, we study the multianalyte/sample capability of our technology as a potential platform to generate a cross‐correlation database via measurement of i) two different ß‐lactams and ii) piperacillin/tazobactam levels in plasma, EBC, saliva, and urine simultaneously on the same chip.

## 2 Methods

The microfluidic biosensor (miLab) was manufactured by using the dry film photoresist (DFR) technology.^[^ ^35^ ^]^ Multiple DFR layers were stacked onto a platinum‐patterned polyimide substrate, on which the microchannels and electrodes are realized (Supporting Information). Each biosensor consists of two consecutive zones; an immobilization area and an electrochemical cell, which are separated by a hydrophobic stopping barrier to prevent electrode fouling.

Sample containing the analyte first goes through the immobilization area by capillary forces, in which competitive binding between the analyte (ß‐lactam in the sample) and ampicillin–biotin conjugate to penicillin binding protein3 (PBP‐3) takes place (Table S2, Supporting Information). Streptavidin–glucose oxidase (Str–GOx) is utilized as a detection enzyme converting glucose to hydrogen peroxide (H_2_O_2_). The signal transduction is achieved through the detection of the generated H_2_O_2_ in the electrochemical cell using a platinum working and counter electrode together with a silver/silver chloride reference electrode. After each incubation step, a washing protocol is employed, where the microchannel was flushed with wash buffer. Unbound biomolecules are removed by applying a vacuum to the channel inlet, without contaminating the measurement cell.

For the electrochemical readout, a glucose solution (40 × 10^−3^ m glucose in 10 × 10^−3^ m phosphate‐buffered saline (PBS)) was pumped through the microfluidic sensor, which is catalyzed on the functionalized surface by GOx. Produced H_2_O_2_ was amperometrically detected at the Pt working electrode by using a fully automated stop‐flow technique for signal amplification (Figures S11 and S12, Supporting Information). Herein, the signal is directly proportional to the amount of immobilized GOx and therefore, inversely proportional to the ß‐lactam concentration in the sample.

The proposed platform relies on electrochemical sensing of particular antibiotics in different mediums, which requires a tailored functionalized surface with high sensitivity. In this study, this was achieved by two key biomolecules, namely PBP‐3 and an ampicillin–biotin conjugate. PBP‐3 was produced in *Escherichia coli* and purified by immobilized metal affinity chromatography. After purification, imidazole (component of the elution buffer) was removed by dialysis, thereby changing the PBP‐3 surface charge distribution and facilitating its immobilization to the channel surface^[^ ^10^ ^]^ (Figures S1 and S2, Supporting Information). The sensitivity of the assay was further improved by using casein as an alternative surface blocker (to previously employed bovine serum albumin) and optimizing each assay component with respect to the incubation time and concentration (Figures S4–S9, Supporting Information). The improved assay yielded a limit‐of‐detection (LOD) of 56 ng mL^−1^ by fitting the measured data points to a four parametric sigmoidal curve (Figure S10, Supporting Information) with a wide operational window up to 1000 µg mL^−1^ and a sample‐to‐result time of less than 90 min. Since our system relies on the detection of analytes with ß‐lactam rings via PBP3, it can be extended for the monitoring of any type of ß‐lactam antibiotic (such as meropenem, cefuroxime) and/or any analyte with ß‐lactam ring (like tazobactam).

For both blood‐based and noninvasive samples, German Landrace hybrid pigs with a weight of 43 ± 3 kg (*n* = 11) were used. Following surgical intervention, a stabilization phase of 15 min, and randomization, piperacillin/tazobactam was injected intravenously with either 200%, 100%, or 50% of the standard dose (4 g piperacillin and 0.5 g tazobactam). Samples of blood, saliva, and urine were taken before (start (ST)), 5 (Baseline (BL)), 30, 60, 120, 180, and 240 min after administration of the antibiotics. Expiratory gas is drawn from the airway's mainstream and cooled down at −7.5 °C for condensation. The EBC samples were collected before injection of antibiotics and 30, 60, 120, 180, and 240 min after (Figure S3, Supporting Information). Whole blood samples were analyzed immediately after collection. Plasma, saliva, EBC, and urine samples were frozen and stored at −80 °C. In the sample preprocessing step, collected raw samples were diluted via a dilution factor optimized for each sample type (Figures S13–S15, Supporting Information). Measured current density was also converted to free drug concentrations to highlight the quantitative nature of the developed strategy by using the calibration curve generated with PBS samples spiked with different concentrations of piperacillin/tazobactam (Figure S10, Supporting Information).

## 3 Results

**Figure 2:**
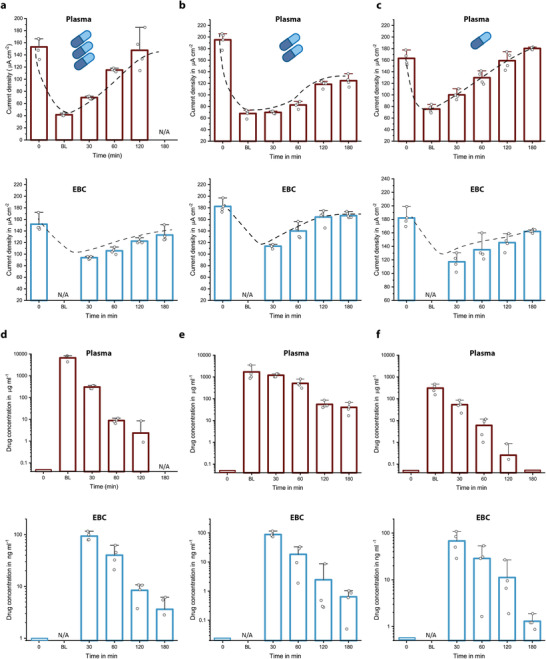
a–f) Measured current densities (a–c) and calculated free drug concentrations (d–f) for plasma and EBC samples of animals given overdose (a,d), normal dose (b,e), and underdose (c,f) piperacillin/tazobactam. A similar clearance behavior and expected concentration decrease with respect to drug dosing regimen were observed for both plasma and EBC measurements over a time period starting from before antibiotic administration (0), after 5 (BL), 30, 60, 120, and 180 min. For EBC measurements, the collection time is 30 min and thus, the first samples were collected at *t* = 30 min. For overdose animal, no plasma sample was collected at *t* = 180 min. Bar plot for *n* = 4 replicates. The error bars represent ±standard deviation (SD).

Utilization of EBC analysis is a promising yet rather unexplored alternative for personalized antibiotherapy. In this study, we first explored the possibility of bypassing transportation related issues by using EBC since the blood–EBC transfer offers a more direct contact compared to other noninvasive alternatives. **Figure** **2** demonstrates the measured course of piperacillin/tazobactam concentrations in plasma and EBC for overdose, normal dose, and underdose scenarios. In all cases, similar clearance behavior (sudden decrease followed by a stepwise increase in current density) was observed for both plasma and EBC measurements. To our best knowledge, this is the first successful demonstration of the detection and clearance of antibiotic concentrations in EBC samples.

**Figure 3:**
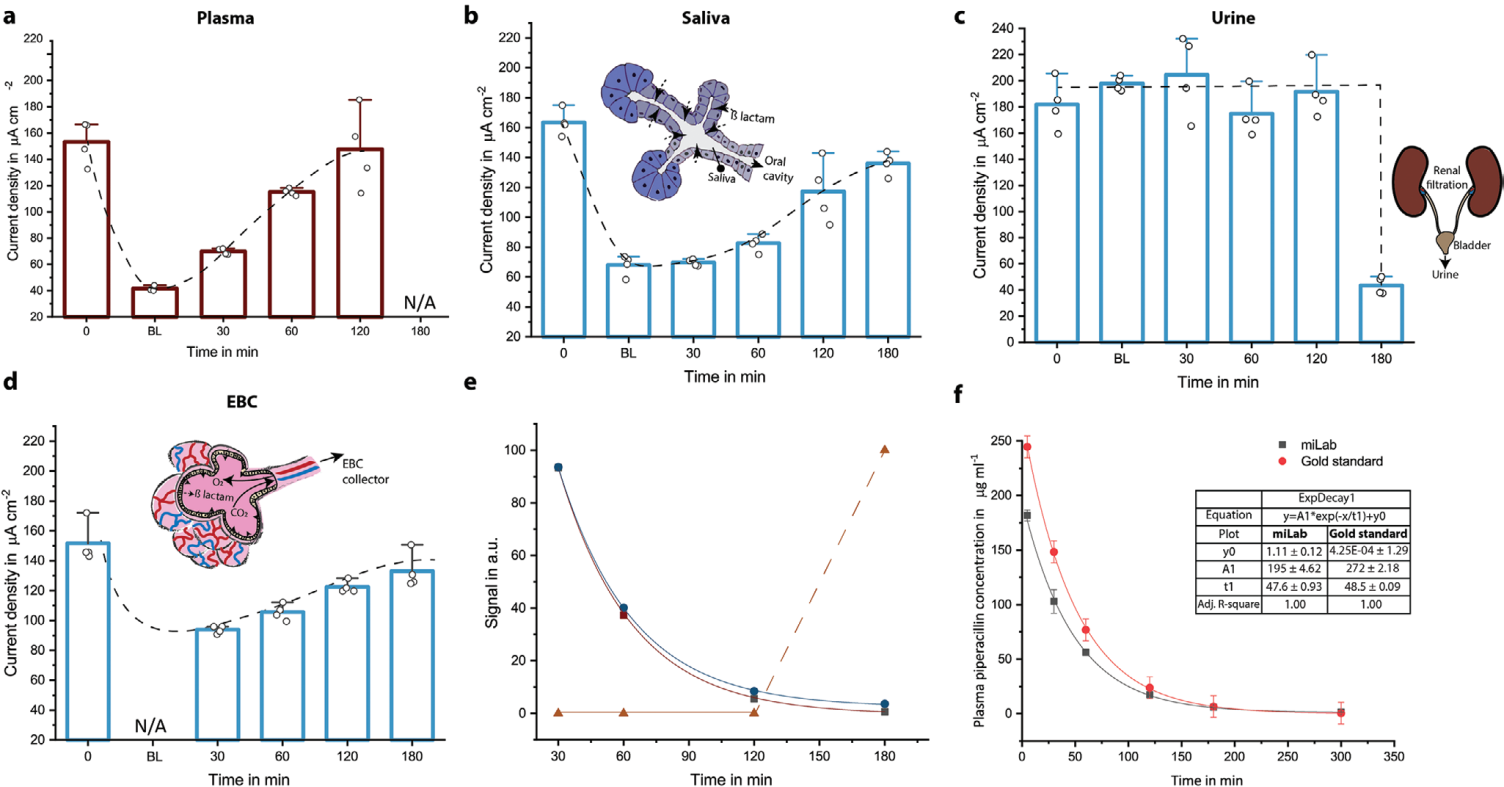
a–d) Demonstration of the measured current densities over a time period starting from before antibiotic administration (0), after 5 (BL), 30, 60, 120, and 180 min for plasma (a), saliva (b), urine (c), and EBC (d) samples of animals given overdose piperacillin/tazobactam. Bar plot for *n* = 4 replicates. Error bars represent ±SD. e) The drug concentration profiles for noninvasive samples revealing the decay in drug concentrations, and f) plasma piperacillin concentration gauged with HPLC measurement (gold standard) and our biosensor platform. Data points are fitted with an exponential decay function to demonstrate clearance behavior (*n* = 7; the error bars represent ±SD).

The measured concentration in plasma immediately after the drug infusion (BL) reflected the dosage regimen (over–normal–under), in average dropping from 6600 to 1700 to 300 µg mL^−1^ (Figure 2d–f). Furthermore, antibiotic clearance over time could be tracked for each individual case (over–normal–under). Nonetheless, there was a striking variance in the rate of drug clearance between the different pigs at a given time. For instance, plasma concentration measured at 60 min was found to be highest for the normal‐dosed animals. EBC measurements further revealed that there is a 4‐order of magnitude decrease in the measured drug concentrations (from 308 µg mL^−1^ to 93 ng mL^−1^ for the overdosed animal, for example), validating the expected concentration drop during the analyte transfer from the blood stream to aerosol particles.^[^ ^36^ ^]^ Interestingly, antibiotic concentrations found at 30 min were consistently around 90 ng mL^−1^. Considering the time it takes to collect breath condensates (also 30 min), this very first sample included the total amount of antibiotics transferred to the aerosols from the beginning of the drug infusion. Since the plasma concentrations after 30 min were found to be significantly different (Figure 2d–f), this upper limit in EBC concentrations may indicate saturation of transport capacity through capillary walls, interstitial space, or epithelial cells (**Figure** **3**d).

Next, we examined the impact of inherent characteristic of different biofluids on the drug clearance behavior by comparing the plasma concentrations with those in EBC, saliva, and urine for an animal given an overdose of piperacillin/tazobactam (Figure 3). The clearance behavior observed in plasma was reflected by both EBC and saliva during the measurement period of 3 h. The rate of change of drug concentrations in EBC and saliva exhibited almost identical trends (Figure 3e). In the case of urine, however, the first antibiotic detection occurred after 3 h, indicating a significant process delay. The striking similarity between EBC and saliva concentration profiles (exponential decay) might be related to how we access the antibiotic carrier medium. Whole saliva was made of secretions from various glands, accumulating within the salivary ducts.^[^ ^1^, ^37^, ^38^ ^]^ Antibiotics passing from the blood stream to the saliva were mixed and diluted within these ducts and then withdrawn from this “chamber.” As a result, the concentration in the saliva reflects the history of drug transportation at a given time giving cumulative (residence time) information rather than instantaneous feedback. In fact, this natural process was mimicked in the EBC collection procedure, where sample collection takes 30 min (Figure S3, Supporting Information). In our opinion, these similarities between measured concentration trends originate from this natural (salivary glands)/artificial (EBC collector) sample collection procedure.

Benchmarking of our system was performed via comparing the plasma piperacillin concentrations measured with HPLC (gold standard) with the ones gauged with our biosensor. Measured concentration values were fitted with an exponential decay function to demonstrate the clearance behavior (Figure 3f and Figure S17 (Supporting Information)). Remarkably similar decay factors “*t*1” were obtained, 47.58 for miLab and 48.55 for gold standard; indicating that both pharmacokinetic characteristics and the plasma levels of piperacillin/tazobactam can be estimated successfully with introduced platform.

**Figure 4:**
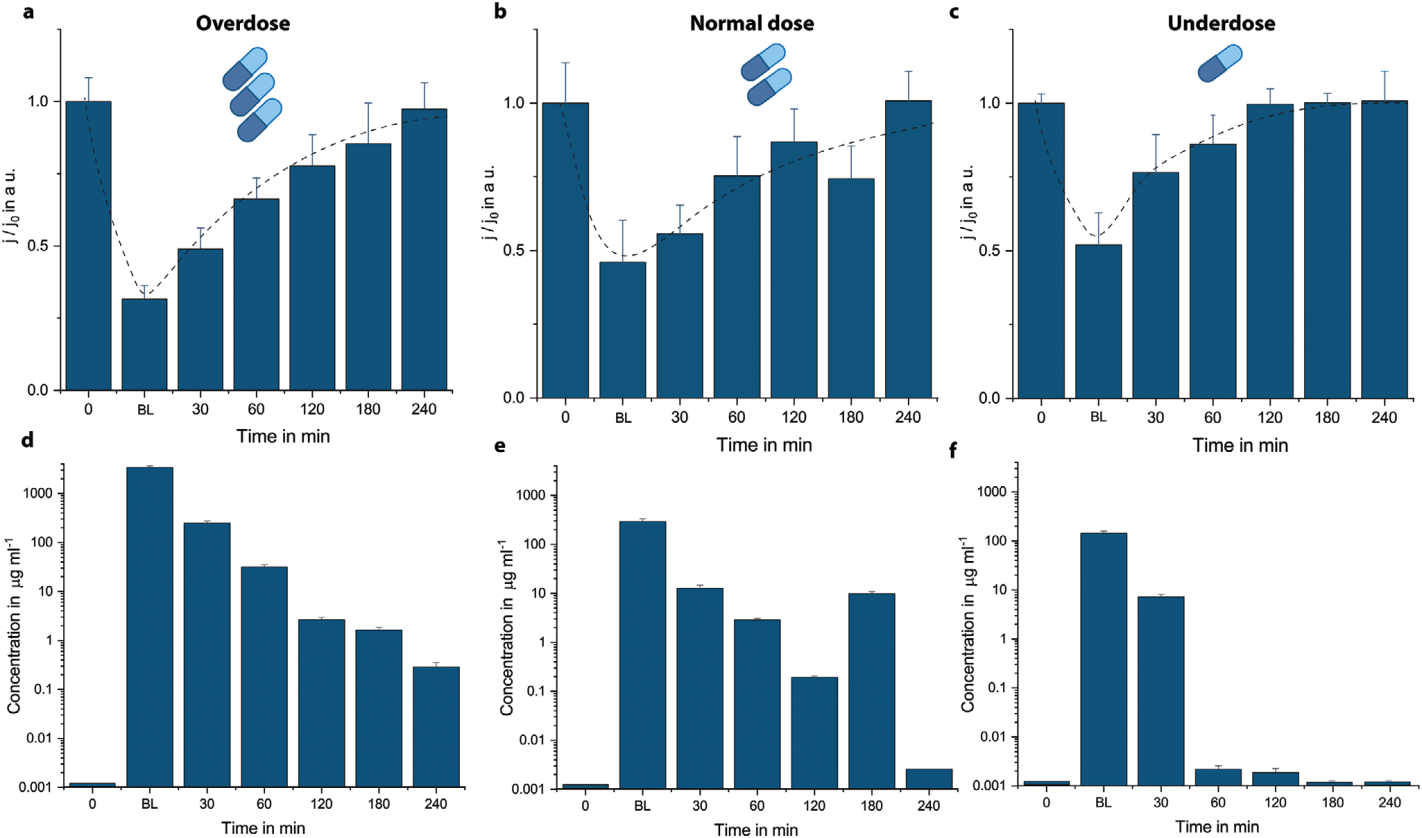
a–f) Measured current densities (a–c) and calculated free drug concentrations (d–f) for untreated whole blood samples of animals given overdose (a,d), normal dose (b,e), and underdose (c,f) piperacillin/tazobactam over a time period starting from before antibiotic administration (0), after 5 (BL), 30, 60, 120, 180, and 240 min. The effects of the drug dosing regimen on drug clearance and measured concentrations were observed. One anomaly was observed during the measurements of normal dosed animals at *t* = 180 min, which was later found to be related to the emergency dosage of anesthetic drug. Bar plot for *n* = 4 replicates. The error bars represent ±SD.

Following the analysis of potential utilization of noninvasive samples in personalized antibiotherapy and benchmarking of our platform, we surveyed the possibility of tracking concentration variations in untreated whole blood samples, with the vision of a TDM platform similar to blood glucometers for POC applications. Blood samples collected from three different pigs given overdose, normal dose, and underdose of ß‐lactam antibiotic were analyzed by using the same microfluidic platform (**Figure** **4**). The applicability of the proposed system could be successfully demonstrated without any matrix effect and with a clearance behavior similar to the plasma measurements (Figure 4a–c). Furthermore, drug concentrations followed the dosage regimen quantitatively. Nonetheless, during the concentration measurements, one anomaly was detected at around 120 min for the normally dosed animal (Figure 4b). This time point correlated with an emergency dosage of propofol (an anesthetic drug) to maintain anesthesia of the animal. An additional test with a whole blood sample spiked with a similar concentration of propofol confirmed the hypothesis of drug interference (Figure S16, Supporting Information).

**Figure 5:**
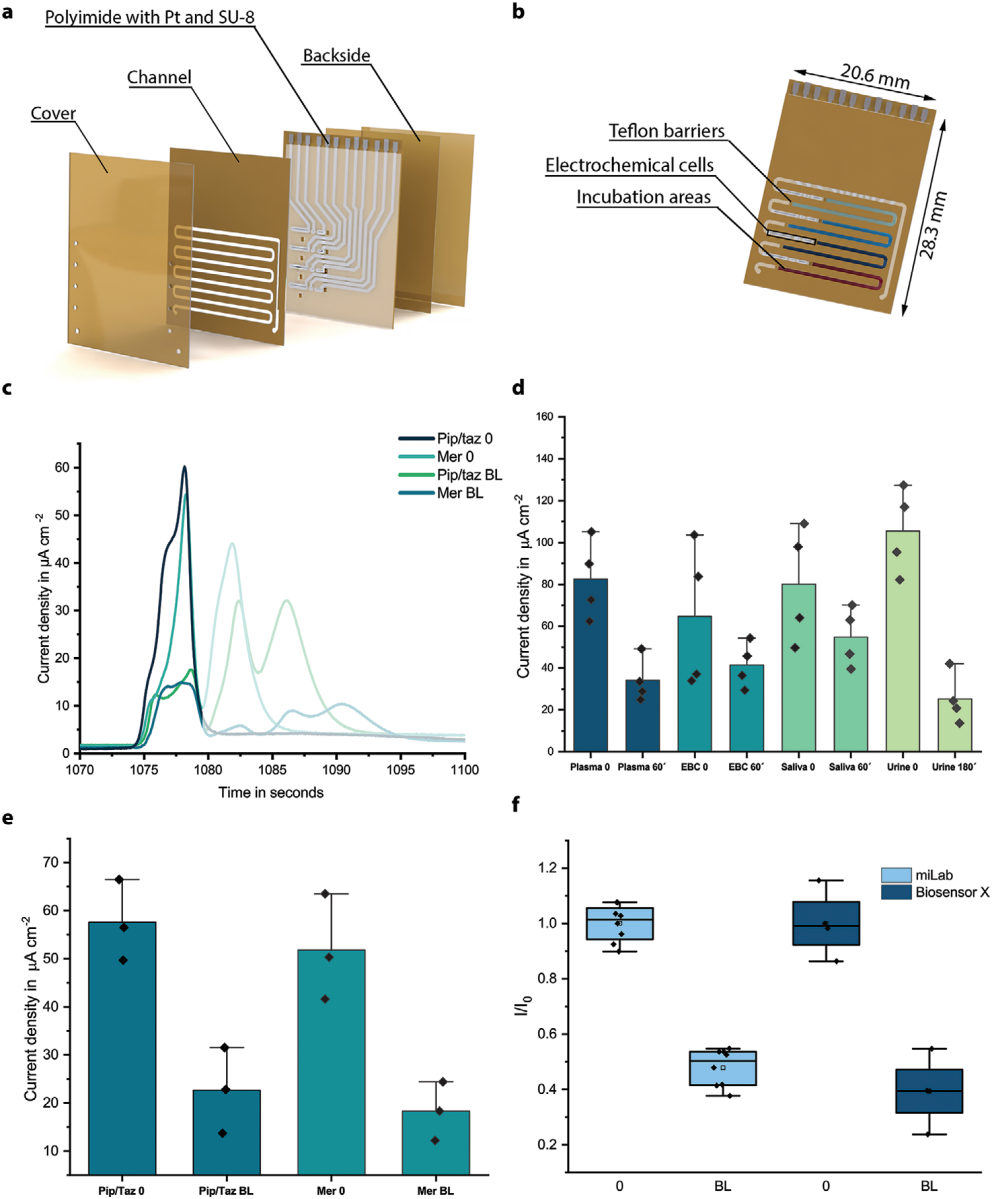
Multianalyte/sample capability of the proposed biosensing technology. a,b) 3D rendering of the stacked multiplexed biosensor (Biosensor X), and four different incubation areas (a), and individual electrochemical cells and Teflon barriers preventing electrode fouling (b). c) Exemplary amperometric signal readout of the multianalyte measurement. The first four successive peaks correspond to the accumulation of electrochemically active species in the immobilization area during stop‐flow protocol. During the “flow” phase, these species are passing through neighboring electrochemical cells in addition to their own individual electrochemical cell, which creates the following faint peaks. d) Demonstration of multisample measurement capability of Biosensor X via temporal evaluation of four different sample types on the same chip. Bar plot for *n* = 4 replicates. The error bars represent ±SD. e) Time‐dependent analysis of two different ß‐lactam in plasma samples of animals given normal dosage of piperacillin/tazobactam and meropenem. Bar plot for *n* = 4 replicates. The error bars represent ±SD. f) Validation of Biosensor X via comparing clearance behavior of piperacillin/tazobactam in plasma samples obtained with Biosensor X and miLab. Box and whisker plot for *n* = 7 (miLab) and *n* = 4 (Biosensor X) replicates. The error bars represent the outlier range.

Observing multidrug interference and its impact on the quantitative analysis, we investigated the multiplexing capability of our technology as a potential platform to generate a cross‐correlation database, which may help to reduce errors in the analysis when interfering analytes/drugs are present in the sample (**Figure** **5**). On the multiplexed biosensor chip (Biosensor X), there are four functionalized zones coupled with their own electrochemical cells (Figure 5a,b).^[^ ^39^, ^40^ ^]^ In Biosensor X, each incubation area is followed by an individual electrochemical cell, resulting in successive peaks by using stop‐flow measurements (Figure 5c). With this architecture, it is possible to combine: i) different assays for multianalyte measurements in a given medium (simultaneous quantification of ß‐lactams and sepsis biomarkers such as inflammation markers), ii) multisample measurements over the same assay (piperacillin/tazobactam analysis in different sample types), or iii) a combination of both.

The possibility of multisample measurement was demonstrated by simultaneously analyzing four different animal samples from pig receiving normal dosage of piperacillin/tazobactam on the same chip (Figure 5d). To demonstrate the clearance behavior of the drug, plasma, EBS, and saliva samples were analyzed before and 60 min after antibiotic administration; urine samples were collected 180 min after antibiotic administration. A clear signal decrease after 60/180 min was observed for each sample, indicating the presence of the drug and its simultaneous detection in different samples using Biosensor X.

To demonstrate the multianalyte sensing capability of Biosensor X, two different ß‐lactams (piperacillin/tazobactam and meropenem) were quantified on the same chip (Figure 5e). Plasma samples of two pigs given normal dosage of piperacillin/tazobactam and meropenem were analyzed and clearance behavior of the antibiotics was demonstrated by measuring the samples before and 5 min after antibiotic administration, simultaneously. To validate clearance behavior of piperacillin/tazobactam obtained with Biosensors X, the same samples were also gauged with our single‐analyte biosensor; where a similar behavior was observed (Figure 5f and Figure S18 (Supporting Information)).

## 4 Discussion

In the current context of clinical TDM, drug concentration measurements are performed by using either chromatographic methods or immunoassays, hence limiting the large scale, distributed TDM practice.^[^ ^1^, ^38^ ^]^ In this regard, our platform offers an opportunity to explore the full potential of personalized antibiotherapy by providing i) a rapid (sample‐to‐result time in less than 90 min) and low‐cost solution for quantitative measurement, ii) information about “free” drug concentration without any sample pretreatment, and iii) the potential for simultaneously measuring different targets without compromising their simplicity. The proposed system is versatile with its wide operational window (measurement range spanning from ng mL^−1^ to µg mL^−1^ with a LOD of 56 ng mL^−1^) and can be used for ß‐lactam antibiotic quantification in different sample types. We benchmarked our system with HPLC measurements (gold standard) via temporal analysis of plasma samples of animal giving normal dosage of piperacillin/tazobactam. Both the measured concentrations and the clearance behavior are in a good agreement with the gold‐standard analysis (Figure 3f and Figure S18 (Supporting Information)).

In a typical electrochemical sensor, biomolecules for signal generation are immobilized on the electrode surface, which requires additional precautions such as protective coating to minimize the fouling caused by complex biofluids.^[^ ^41^ ^]^ In our system, we inherently bypass the fouling issue, as we separated the immobilization zone and the electrochemical cell with a hydrophobic barrier (Figure 1 and Figure S11 (Supporting Information)). This design strategy enables us to work with complex biofluids such as whole blood without compromising sensitivity. We also tested the possibility of measuring i) complex biofluids and ii) different analytes on the same chip simultaneously by using our multiplexed chip, Biosensor X. The results obtained were validated with our single‐analyte biosensor and suggest that simultaneous analysis of different samples and various drugs can be achieved by our multiplexed chip design.

Our observations reveal that there are distinct clearance behaviors for different mediums in accordance with their complex transport mechanisms. In principle, blood–EBC antibiotic transfer is expected to be more direct through capillary walls densely surrounding alveoli.^[^ ^30^ ^]^ This potential of instantaneous access, however, is very difficult to realize in practice. If the exhaled breath condensate is collected in an external cooled chamber over a period of time, which was the case in our study, the accessible information from EBC involves a time delay and a history of concentration changes. As a remedy, alternative strategies can be utilized such as wearable breath sensors including face masks, in‐mouth/in‐nose implants, or augmented sensing platforms exploiting natural sensors in the respiratory tract.^[^ ^42^ ^]^ In this case, however, the sensor should be sensitive and selective enough to detect the analyte within more than 3000 volatile organic compounds in the presence of other exogenous effects.^[^ ^1^ ^]^ Therefore, in our opinion, the near‐future potential of exhaled breath for personalized antibiotherapy lies in multisample framework, providing additional insights into metabolic activities. In the light of the knowledge acquired in this work, one of our future work will be the extension of our paper‐based wearable sensor,^[^ ^43^ ^]^ which can be integrated onto any type of face masks, for the real‐time and continuous measurement of ß‐lactam antibiotics from exhaled breath.

Transport dynamics into saliva glands depend on the dissociation constant, lipophilicity, pH, protein binding affinity, and ionizability of the drug,^[^ ^1^, ^38^ ^]^ and thus can be much more complex than capillary diffusion through alveoli.^[^ ^37^, ^44^ ^]^ Our saliva and EBC measurements yielded a similar exponential decay (Figure 3e), indicating that piperacillin/tazobactam transfer from blood to collected saliva was not influenced significantly by these inherent complexities. This outcome shows the potential of our sensor for personalized saliva‐based ß‐lactam monitoring. Urine goes through an even more complicated cycle, which composes a very rich sample containing urea, creatinine, ammonia, uric acid, blood cells, hormones, bilirubin, amino acids, proteins, sulfate, phosphate, chloride, sodium, potassium, and other trace elements.^[^ ^45^ ^]^ Therefore, urine analysis is typically prone to low signal‐to‐noise ratio due to the matrix effect. In our platform, we alleviated this issue by working with diluted urine samples, for which the assay sensitivity was optimized to be functional at very low concentrations. During the experiments, we did not observe any decrease in the current density for the first five measurements, which is followed by a sharp decrease indicating the presence of ß‐lactam in urine (Figure 3e).

Another important use case for the developed sensor is the whole blood measurement, which enables an easy access to pharmacokinetically relevant information such as interpatient variance, effect of external factors, and dosage. The success of the antibiotherapy heavily depends on keeping the blood antibiotic concentrations within the therapeutic range and this range must be tailored to respond the patient's unique PK/PD. Such an individualization process, however, requires frequent sampling. Herein, low volume requirement and the ability to process untreated whole blood with the proposed sensor may catalyze the realization of on‐site TDM. This is of particular importance for specific patient groups like pediatric, neonatal, and elderly patients, for whom repetitive blood collection via venipuncture is difficult. With a further improvement of the design and integrating all necessary components in one handheld device, it could be possible to utilize our platform for decentralized TDM, similar to the diabetes monitoring via blood glucometer.

Alternative samples offer a great potential for a wide range of future on‐site TDM applications. In the clinical practice, however, there are many uncertainties regarding the diagnostic correspondence of the measured concentrations in noninvasive samples and how these concentrations are correlated to the more familiar blood‐based counterparts. Unfortunately, a direct correlation between a noninvasive sample and blood for a given analyte (piperacillin/tazobactam) is hard to formulate mainly due to the nonlinear transport mechanisms, which is further complicated by interpatient variance and exogenous factors. Our observations demonstrate that we need to include more animals in our study to create a “database,” which may reveal the unknown link between blood and noninvasive matrices. This study could also be supported in the future with a prospective and observational pharmacokinetic study in patient populations. Consequently, multiplexed sensing can help to improve the overall reliability of the system by providing a physiological information for active calibration and correction of target concentrations.^[^ ^38^, ^46^ ^]^ Therefore, any proposed remedy has to be simple, fast, and economical enough to make therapeutic drug management decentralized.

In this work, we responded to this call by implementing a versatile platform that can operate with multianalyte/sample tasks. A successful realization of either blood‐based or noninvasive on‐site monitoring of antibiotics using such a biosensor could be a game‐changer in the antibiotherapy in the longer run and beyond, since this technology could be extended to measure other drugs and biomarkers.^[^ ^47^ ^]^ For instance, combining TDM of antibiotics and inflammation progress biomarkers could pave the way to personalized antibiotherapy.^[^ ^48^, ^49^ ^]^ This could be a significant landmark on the global combat against antibiotic resistances.

## 5 Experimental Section

Details of the experiments and methods are described in the Supporting Information.

## Conflict of Interest

The authors declare no conflict of interest.

## Supporting information

Supporting information

## Data Availability

Research data are not shared.
