# Supplementary material for: Biosensor‐Enabled Multiplexed On‐Site Therapeutic Drug Monitoring of Antibiotics
Source: Adv Mater. 2021 Sep 21;34(2):2104555. doi: 10.1002/adma.202104555 (PMC11468941; doi:10.1002/adma.202104555)
Supplement: Supplementary file 1 — Supporting information [file ADMA-34-2104555-s001.pdf]

© 2021 Wiley-VCH GmbH

ADVANCED MATERIALS

Supporting Information

for *Adv. Mater.,* DOI: 10.1002/adma.202104555

Biosensor-Enabled Multiplexed On-Site Therapeutic Drug Monitoring of Antibiotics

*H. Ceren Ates, Hasti Mohsenin, Christin Wenzel, Regina T. Glatz, Hanna J. Wagner, Richard Bruch, Nico Hoefflin, Sashko Spassov, Lea Streicher, Sara Lozano-Zahonero, Bernd Flamm, Rainer Trittler, Martin J. Hug, Maja Köhn, Johannes Schmidt, Stefan Schumann, Gerald A. Urban, Wilfried Weber, and Can Dincer**

Supporting Information

Biosensor-Enabled Multiplexed On-Site Therapeutic Drug Monitoring of Antibiotics

H. Ceren Ates, Hasti Mohsenin, Christin Wenzel, Regina T. Glatz, Hanna J. Wagner, Richard Bruch, Nico Hoefflin, Sashko Spassov, Lea Streicher, Sara Lozano-Zahonero, Bernd Flamm, Rainer Trittler, Martin J. Hug, Maja Köhn, Johannes Schmidt, Stefan Schumann, Gerald A. Urban, Wilfried Weber, Can Dincer*

H. Ceren Ates, Regina Glatz, Richard Bruch, Can Dincer
University of Freiburg, FIT Freiburg Center for Interactive Materials and Bioinspired Technologies, Georges-Koehler-Allee 105, 79110 Freiburg, Germany
E-mail: dincer@imtek.de

H. Ceren Ates, Regina T. Glatz, Richard Bruch, Gerald A. Urban, Can Dincer
University of Freiburg, Department of Microsystems Engineering (IMTEK), Laboratory for Sensors, Georges-Koehler-Allee 103, 79110 Freiburg, Germany

Hasti Mohsenin, Hanna J. Wagner, Nico Hoefflin, Maja Köhn, Wilfried Weber
University of Freiburg, Faculty of Biology and Signalling Research Centres BIOSS and CIBSS, Schaenzlestrasse 18, 79104 Freiburg, Germany

Hanna J. Wagner
ETH Zurich, Department of Biosystems Science and Engineering, Mattenstrasse 26, 4058 Basel, Switzerland

Christin Wenzel, Sashko Spassov, Lea Streicher, Sara Lozano-Zahonero, Bernd Flamm, Johannes Schmidt, Stefan Schumann
Medical Center – University of Freiburg, Department of Anesthesiology and Critical Care, Faculty of Medicine, Hugstetter Str. 55, 79085 Freiburg, Germany

Rainer Trittler, Martin J. Hug
Medical Center – University of Freiburg, Department of Pharmacy, Hugstetter Straße 55, 79106 Freiburg

Gerald A. Urban
University of Freiburg, Freiburg Materials Research Centre (FMF), Stefan-Meier-Straße 21, 79104 Freiburg, Germany

1. Chip design and fabrication

A polyimide foil, Pyralux® AP8525R (DuPont, USA) was utilized after 1 h of copper etching as a substrate material for the biosensor fabrication[1]. The platinum (Pt) electrode structures were prepared by using lift-off technology. The negative tone photoresist, ma-N 1420 (Micro Resist Technology, Germany), was spin coated onto the substrate at 3,000 rpm for 30 s, exposed to UV (ultraviolet) light on an exposure unit (Hellas, Bungard Elektronik, Germany) and developed. Pt electrodes (200 nm) were deposited via physical vapor deposition in a cleanroom environment. SU-8 3005 (MicroChem Corp., USA) layer (5 µm thick) was applied by spin coating at 4,000 rpm for 30 s to (i) precisely define the electrode areas, (ii) electrically isolate electrodes, (iii) realize small wells to implement stopping barriers later on. After UV light exposure and development steps, the SU-8 coated wafer was hard-baked in an oven (Binder, Germany) for 3 h at 150 °C.

After hard-bake, in order to remove possible SU-8 residues on the Pt electrodes, a low frequency oxygen plasma (Tetra-30-LF-PC, Diener, Germany) was applied for 3 min at 300 W at room temperature. Ag/AgCl on-chip reference electrode was realized by galvanic deposition in an Arguna S solution (Umicore Galvanotechnik, Germany) employing a constant current of −0.2524 mA for 20 min with a silver wire as counter electrode. After chlorination of the silver layer in a 0.1 M Potassium chloride (KCl) solution employing a constant current of +135.44 µA for 10 min, the reference electrode was finalized.

The microfluidic channels were realized by stacking dry film photoresist layers, Pyralux® PC1025 (DuPont, USA), on the platinum patterned polyimide substrate. DFR layers were first exposed for 50 s to UV light and then developed in a 1% sodium carbonate (Na_2CO_3) solution at 42 °C in an ultrasonic bath (Sonorex Super 10 P, Bandelin, Germany). To prevent any over-development, DFR layers were subsequently immersed into a 1% HCl bath for 1 min.

Hydrophobic stopping barrier, which separates the electrochemical cell from the immobilization area, were formed by dispensing small drops of 3% Teflon® (AF 1600, DuPont, USA) into small SU-8 wells. Finally, the developed layers were aligned on the substrate and fixed using a laminator (HRL 350, Ozatec). After lamination, the wafer was diced into individual biosensor chips and hard-baked at 160 °C for 3 h with a ramp function. Visual demonstration of chip fabrication, on-chip assay incubation and the measurement procedure can be found[1].

2. Cost estimation of the biosensor used in this study

Fabrication- and functionalization-related costs of the biosensor, were calculated by using the in-house used container sizes and prices of each step. As these prices are based on relatively small container sizes, the price will strongly vary, depending on the quantity of manufactured biosensor chips. Calculations were made with a batch of 4 wafers, each containing 130 biosensors[2].

Table S1: Estimation of the costs for the fabrication and functionalization of a single biosensor.

No.	Step	Reagent/Material used	Cost/biosensor
1)	Substrate preparation	Pyralux AP8525	0.045 €
2)	Lift-off for Platinum deposition	ma-N 1420	0.024 €
3)	Platinum deposition	Pt PVD	0.480 €
4)	Electrode isolation	Su-8 3005	0.067 €
5)	Reference electrode	Ag/AgCl	<< 0.001 €
6)	Dry film photoresist	Pyralux PC1025	0.017 €
7)	Surface functionalization	Penicillin binding protein	<< 0.001 €
8)	Blocking of the free surface sites	Casein	<< 0.001 €
9)	Competitor molecule	Ampicillin-biotin	<< 0.001 €
10)	Signal molecule	Streptavidin GOx	0.010 €
		**Sum of the functionalized biosensor chip**	**0.644 €**

3. Production and purification of the penicillin-binding protein 3 (PBP-3)

The PBP-3 bacterial expression vector was transformed into E. coli BL21 (DE3) pLysS (Invitrogen).[3] Bacteria were grown in LB medium (Roth) containing 100 µg mL^{-1} ampicillin (Roth) and 36 µg mL^{-1} chloramphenicol (Roth) at 37 °C, 150 rpm. Once OD600 = 0.6 was reached, protein production was induced with 1 mM isopropyl ß-D-1-thiogalactopyranoside (IPTG) for 14 h at 20 °C. Cells were harvested by centrifugation at 6,000 × g for 10 min and the bacterial pellets were resuspended in Ni-Lysis buffer (50 mM NaH_2PO_4, 300 mM NaCl, 10 mM imidazole, pH 8.0) and lysed by sonication (60% amplitude, 0.5 s/1 s pulse/pause intervals using a Bandelin Sonoplus HD 3100 homogenizer, Germany, for 10 min on ice). Cellular debris was removed by centrifugation at 30,000 × g for 30 min at 4 °C and the supernatant was purified by gravity flow Ni^{2+}-NTA chromatography using superflow agarose (QIAGEN, Germany) following the manufacturer's instructions. The buffer of the purified proteins was exchanged by dialysis against 5 L Ni-Lysis buffer without imidazole overnight, using SnakeSkin dialysis tubes with 3.5k

MWCO (Thermo Fisher Scientific, USA). The purified protein was analyzed by SDS-PAGE (12% (w/v) gels) and Coomassie brilliant blue staining (**Figure S1**). The protein concentration was determined by Bradford method (Bio-Rad, USA) with bovine serum albumin (Carl Roth GmbH + Co. KG, Germany) as standard. Proteins were supplemented with 10% glycerol and concentrated to a final concentration of 10 mg mL^{-1} using Spin-X UF 6 (10k MWCO, Corning Inc.,USA). Aliquots were stored at –80 °C and thawed for each experiment.

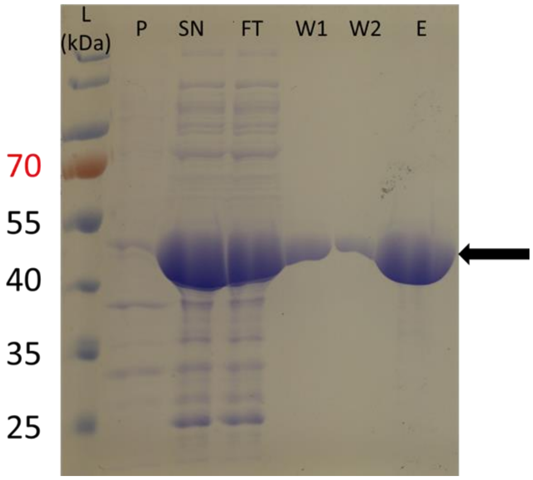

Figure S1: Analysis of PBP-3 purification by SDS-PAGE. 6 µL of PageRuler Prestained Protein Ladder (Thermo Fischer Scientific, USA, lane 1) and 10 µL of samples were separated on a 12% (w/v) SDS-gel. P, pellet; SN, supernatant; FT, flow-through; W1, wash fraction 1; W2, wash fraction 2; E, elution. The arrow indicates the purified PBP-3 protein (expected size: 42 kDa).

4. Synthesis of the ampicillin-biotin conjugate

1 mg ampicillin sodium salt (Carl Roth GmbH + Co. KG, Germany) was dissolved in 842 µl dried ethanol (max. 0.01% H_2O, Merck KGaA, Germany). NHS-PEG$_{24}$-biotin (Sigma-Aldrich, Germany, QBD10774) was dissolved in anhydrous DMSO (Sigma-Aldrich, Germany) under continuous Argon flow in a desiccator to a final concentration of 50 mg mL^{-1}. 158 µL NHS-PEG$_{24}$-biotin solution (5.4 µmol) was mixed with 842 µL ampicillin solution (2.7 µmol) and incubated at RT for 3 h while shaking (400 rpm)[3]. The reaction characterization was carried out by MALDI-MS. Conjugates were aliquoted under argon flow and stored at –80 °C. The product

was used without further purification as the non-reacted NHS-PEG$_{24}$-biotin was eliminated by washing after binding of the ampicillin-biotin conjugate to PBP-3 on the biosensor.

5. Characterization of the biomolecules

Samples were dissolved in acetonitrile (HPLC grade, Sigma-Aldrich, Germany). 5 µl of the sample solution was mixed with 5 µl of a saturated solution of α-cyano-4-hydroxycinnamic acid matrix for MALDI-MS (Sigma-Aldrich, Germany) in acetonitrile. 1.5 µl of the latter mixture was applied to the MALDI target and let dry. Analysis was carried out on a Microflex MALDI mass spectrometer (Bruker, USA) and data was processed via mMass software.[4]

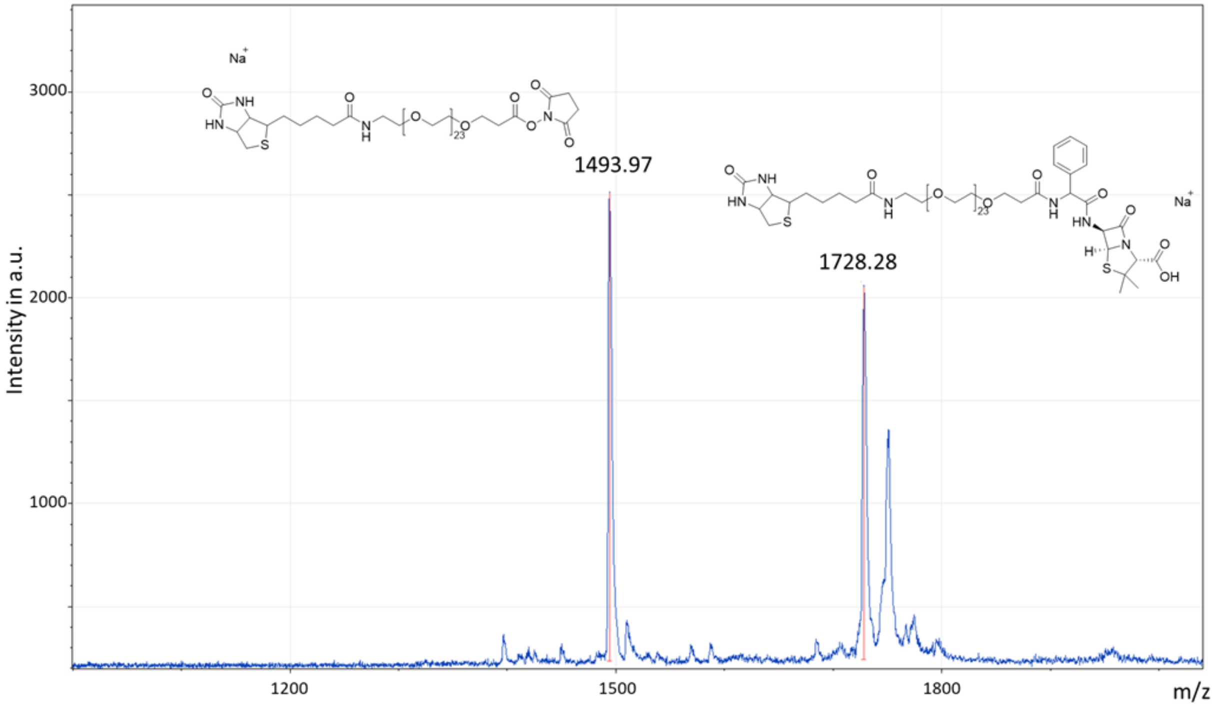

Figure S2: Time-of-flight MALDI spectra analysis. The marked left peak corresponds to the excess amount of reactant (NHS (PEG)24-biotin) with an expected molecular weight of ~1493 g mol^{-1}. The marked right peak corresponds to final product (ampicillin-(PEG)24-biotin) with an expected molecular weight of ~172 g mol^{-1}.

6. Animal studies

The study was approved by the review board of local ethical committee (Regierungspräsidium Freiburg; Ref.-No. G-20/45) and performed in accordance with the European and German law on protection of animals used for scientific purposes. With the intention to reduce animal experiments, this and another study regarding respiratory system mechanics during mechanical ventilation, not interfering with each other were conducted with the same animals.

German Landrace hybrid pigs with a weight of 43 ± 3 kg (n=11) were used for this study. After a fasting period of 12 h, animals were premedicated with an intramuscular injection of ketamine (20 mg kg^{-1}) and midazolam (0.5 mg kg^{-1}). Anaesthesia was induced intravenously (i.v.) with propofol and maintained with a continuous infusion of midazolam (0.3 mg kg^{-1} h^{-1}), ketamine (10 mg kg^{-1} h^{-1}) and dexmedetomidine (1-5 µg kg^{-1} h^{-1}). To prevent dehydration during ventilation Ringer solution (10 ml kg^{-1} h^{-1}; B. Braun Melsungen AG, Germany) was infused intravenously. The pigs were tracheally intubated with a standard endotracheal tube with an inner diameter of 7.5 mm and ventilated with an intensive care ventilator (Evita V500, Draeger Medical, Germany) with a tidal volume of 7 ml kg^{-1}, inspiratory oxygen fraction of 0.3 and a positive end-expiratory pressure of 5 cm H_2O. Respiratory rate was adjusted to achieve an End-Tidal CO_2 of 35-45 mm Hg. An arterial catheter (4F, Pulsion Medical Systems, Germany) was placed in the femoral artery for taking blood samples. A urinary catheter was inserted into the bladder for taking urine samples. Following surgical intervention and a stabilization phase of 15 min and randomization, piperacillin/tazobactam was injected intravenously with either 200%, 100% or 50% of standard dose (4 g piperacillin/tazobactam 0.5 g).

Samples of blood and urine were taken before (ST), and 5 (BL), 30, 60, 120, 180 and 240 min after injection of antibiotics. Additionally, saliva probes were drawn via an intravenous cannula (Vasofix 14G, B. Braun Melsungen AG, Germany) before (ST), 5 (BL) 30, 60, 120 and 180 min after injection of antibiotics.

To collect exhaled breath condensate (EBC), expiratory gas was drawn at a flow rate of 180 ml min^{-1} using a flow generator (Vamos, Dräger Medical, Lübeck, Germany) from the airway's mainstream measurement for 30 min. The gas was cooled down for condensation at -7.5 °C. EBC samples were taken before injection of antibiotics and after 30, 60, 120 and 180 minutes.

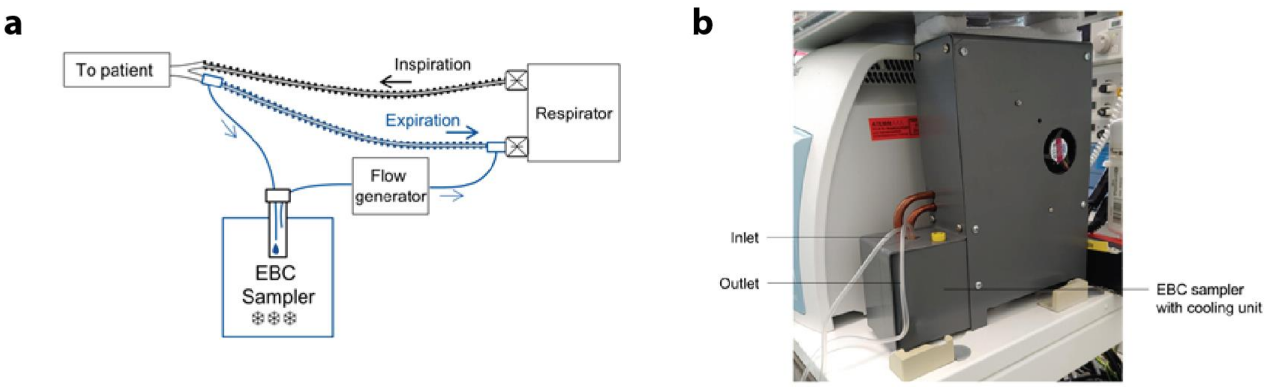

Figure S3: (a) Schematic drawing of setup to measure exhaled breath condensate (EBC). Via side stream measurement 180 ml min^{-1} was continuously drawn from patients' exhaled air using a flow generator (Vamos, Dräger Medical, Lübeck, Germany) and cooled down for condensation at –7.5 °C. (b) Illustration of the device for sampling EBC from patients' exhaled air using side stream measuring.

7. Statistical analysis and calculation of limit of detection

All data were expressed as mean ± standard error with n = 4. For the calculation of the limit of detection (LOD), the fitted calibration curve was used to determine the signal response from the sensor at the LOD, using the following equation 1:

$$y = LOD_{signal} = Signal_{blank} - 1.654 * SD_{blank} - 1.654 * SD_{lowest\_measured\_concentration} \qquad \text{(Eq. 1)}$$

To calculate the responding concentration of the obtained LOD_{signal}, the equation of the 4-parametric logistic fit is needed; equation 2:

$$y = A_{min} + \frac{A_{max} - A_{min}}{1 + (\frac{x}{x_0})^p} \qquad \text{(Eq. 2)}$$

where A_{min} is equal to the lower saturation plateau for higher concentrations, A_{max} corresponds to the higher saturation plateau for very low concentrations. x_0 describes the concentration value for which 50% of the signal is present and p is the slope of the fitting curve in the dynamic region. By placing the obtained LOD_{signal} of equation 1 into equation 2, the LOD can be calculated:

$$LOD_{concentration} = x_0 * (\frac{LOD_{signal} - A_{max}}{A_{min} - LOD_{signal}})^{\frac{1}{p}} \qquad \text{(Eq. 3)}$$

These calculations were performed for every calibration curve, shown in this publication.

8. On-chip assay optimization

On-chip incubation started with PBP-3 adsorption by 1-hour incubation of the channel surface with 250 µg ml^{-1} PBP-3 (**Table S2**). Then, active surface sites were blocked with biotin-free casein (85R-108, Fitzgerald, USA) for 20 minutes. The third step of the assay relied on the competitive binding of piperacillin in the sample solutions with different concentrations and 0.2 µg ml^{-1} biotinylated ampicillin to the PBP-3. Finally, 10 µg ml^{-1} Streptavidin-glucose oxidase (FGI-65R-S125, Biozol, Germany) was utilized with a 15-minute incubation for the signal generation. After each incubation step, a washing protocol was employed, where the microchannel was flushed with wash buffer (50 µl of 0.05% TWEEN 20 in 10 mM PBS, Sigma Aldrich, USA). Unbound biomolecules were removed by applying a vacuum to the channel inlet, without contaminating the measurement cell. Conversion of glucose to hydrogen peroxide by streptavidin-glucose oxidase resulted in an electrical current through the measurement of H_2O_2 in the electrochemical cell, followed by the signal readout.

Table S2: Incubation protocol of all assay components employed for the on-chip antibiotic assay.

No.	Assay component	Concentration	Incubation time
1)	Penicillin binding protein (PBP-3)	250 µg ml^{-1}	60 min
2)	Casein	1%	20 min
3)	Ampicillin-biotin<br>Piperacillin/tazobactam	0.2 µg ml^{-1}<br>Drug in the sample	60 min
4)	Streptavidin Glucose oxidase	10 µg ml^{-1}	15 min
		**Total assay time**	155 min

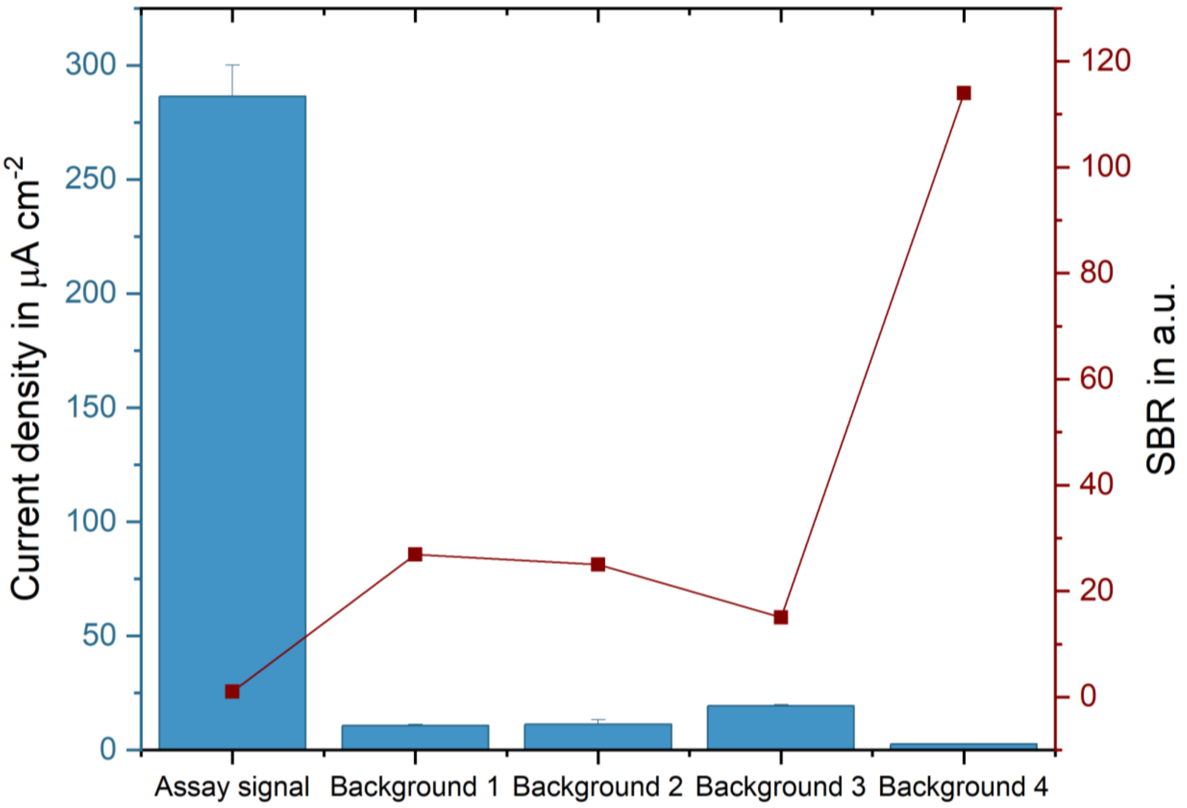

Figure S4: Determination of non-specific binding of assay components. Background 1: The signal obtained by 10 µg ml^{-1} Streptavidin-GO_x immobilized on the surface blocked with casein. Background 2: The signal obtained by the channel functionalized with 250 µg ml^{-1} PBP-3, blocked with casein and incubated with 10 µg ml^{-1} Streptavidin-GO_x. Background 3: The signal obtained by the channel blocked with casein, and incubated with 10 µg ml^{-1} ampicillin-biotin and 10 µg ml^{-1} Streptavidin-GO_x. Background 4: The signal obtained by the channel functionalized with 250 µg ml^{-1} PBP-3, blocked with casein and incubated with 10 µg ml^{-1} ampicillin-biotin. The signal to background ratio (SBR) was calculated by dividing the individual mean values of the signal by the mean values of the individual backgrounds. Error bars represent ± SD of n = 4 replicates.

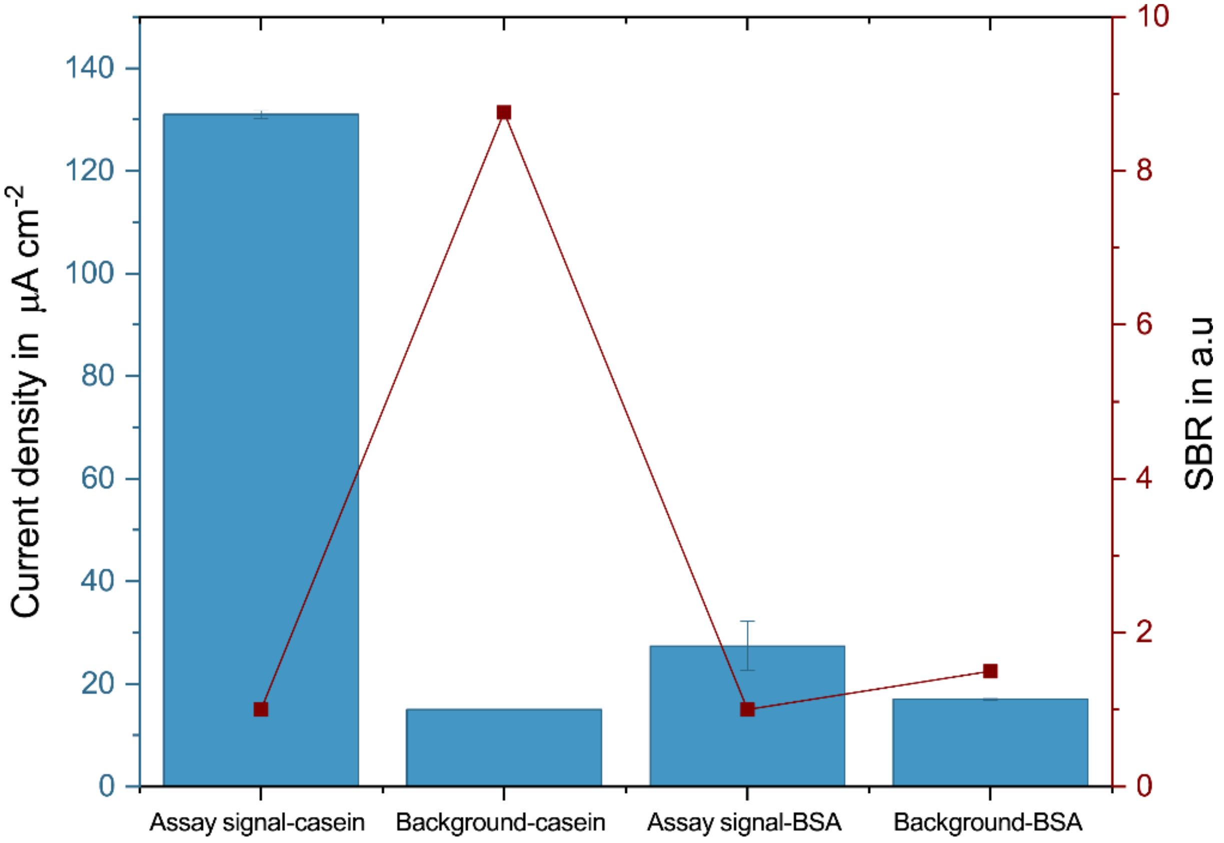

Figure S5: Comparison of two different blocking strategies. The signal to background ratio was calculated by dividing the mean values of the signal by the mean values of the individual backgrounds of casein and BSA. Error bars represent ± SD of n = 4 replicates.

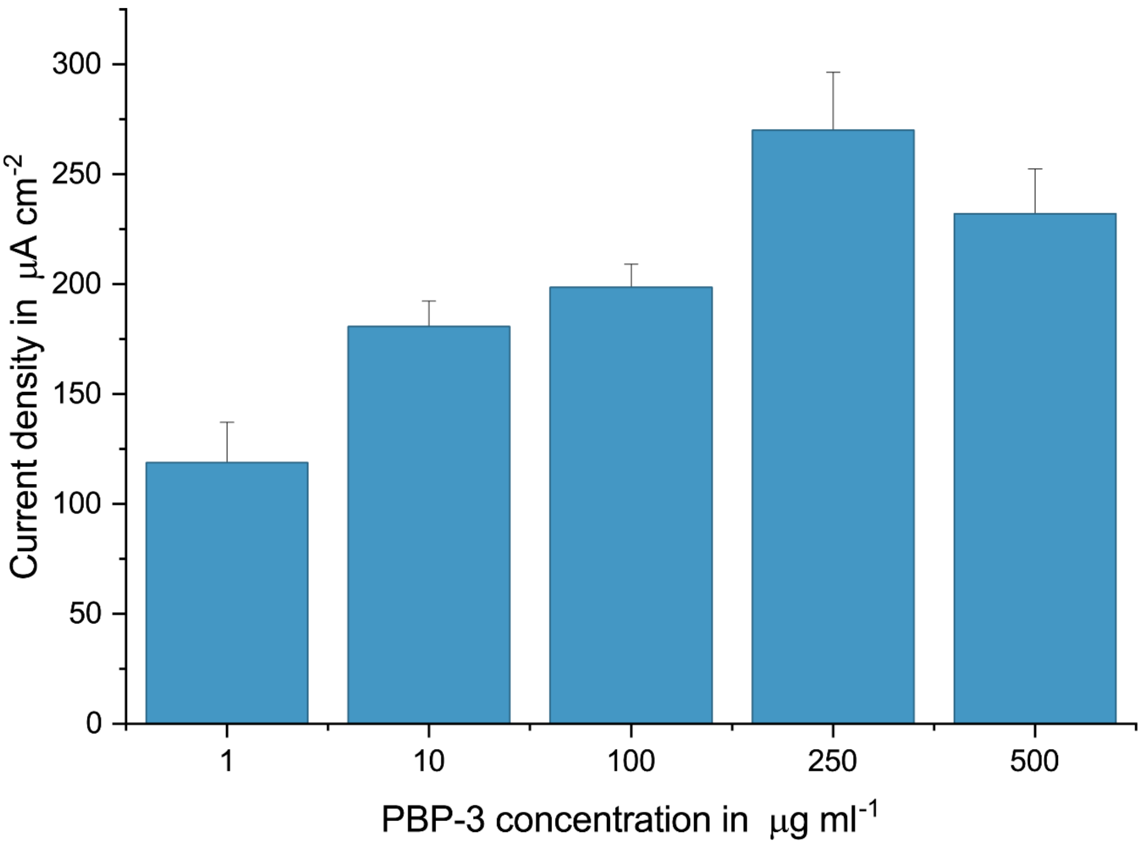

Figure S6: Comparison of different PBP-3 concentrations to functionalize the surface of the chip. For ß-lactam measurements, unless otherwise stated, the concentration of PBP-3 was selected as 250 µg ml^{-1}. Error bars represent ± SD of n = 4 replicates.

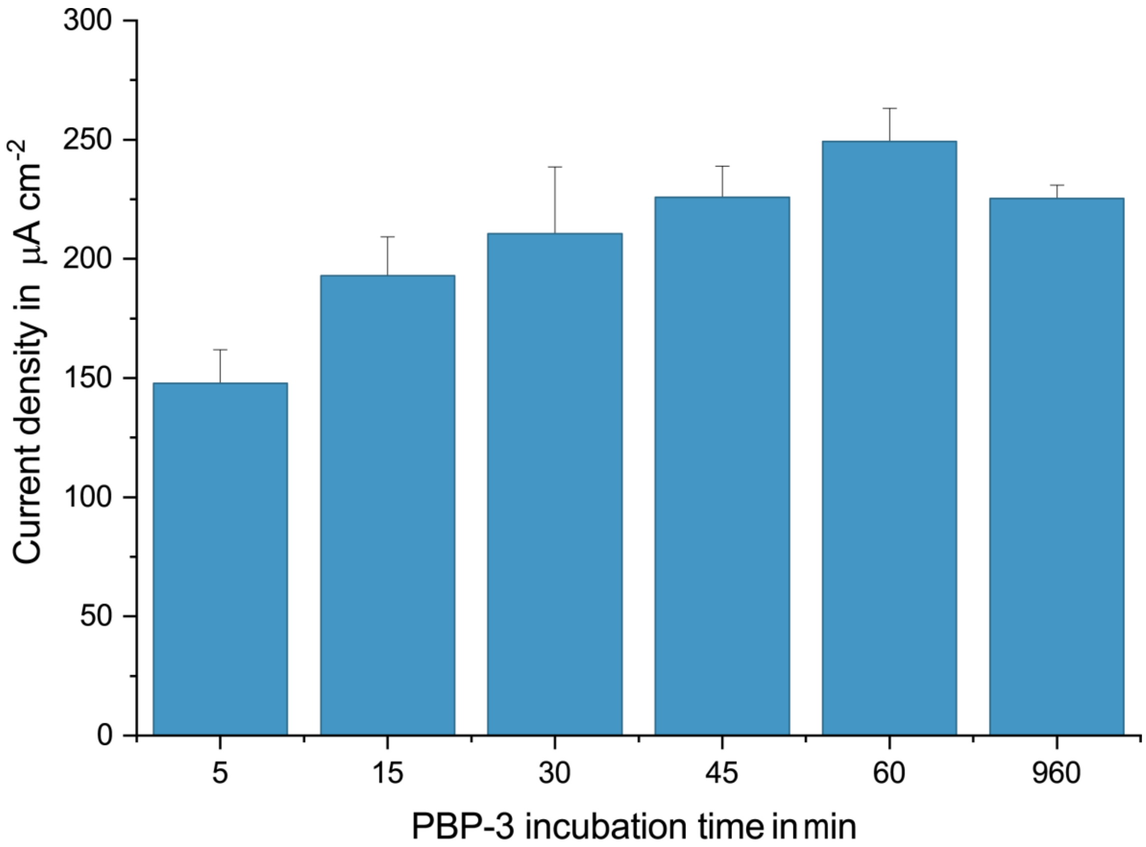

Figure S7: Comparison of different PBP-3 (250 µg ml^{-1}) incubation times for the functionalization of the chip surface. For ß-lactam measurements, unless otherwise stated, the incubation time with PBP-3 was selected as 1 hour. Error bars represent ± SD of n = 4 replicates.

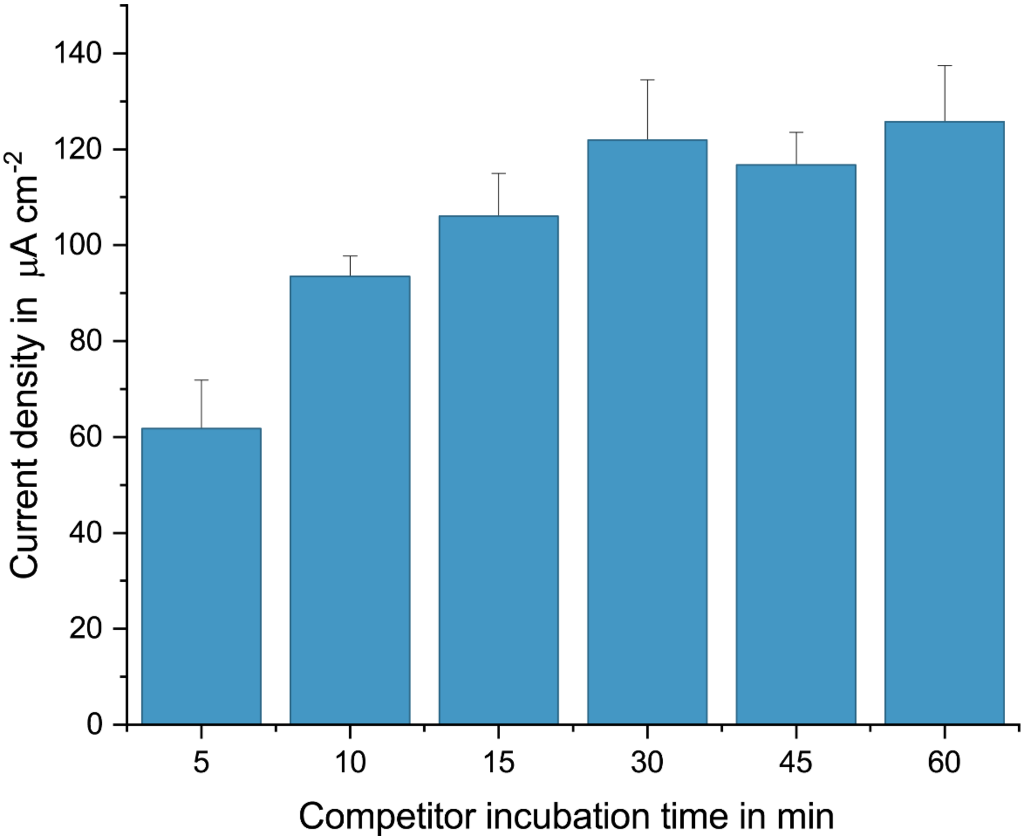

Figure S8: Comparison of different ampicillin-biotin (10 µg ml^{-1}) incubation times. For assay measurements, unless otherwise stated, the competitor incubation time was selected as 1 hour. Error bars represent ± SD of n = 4 replicates.

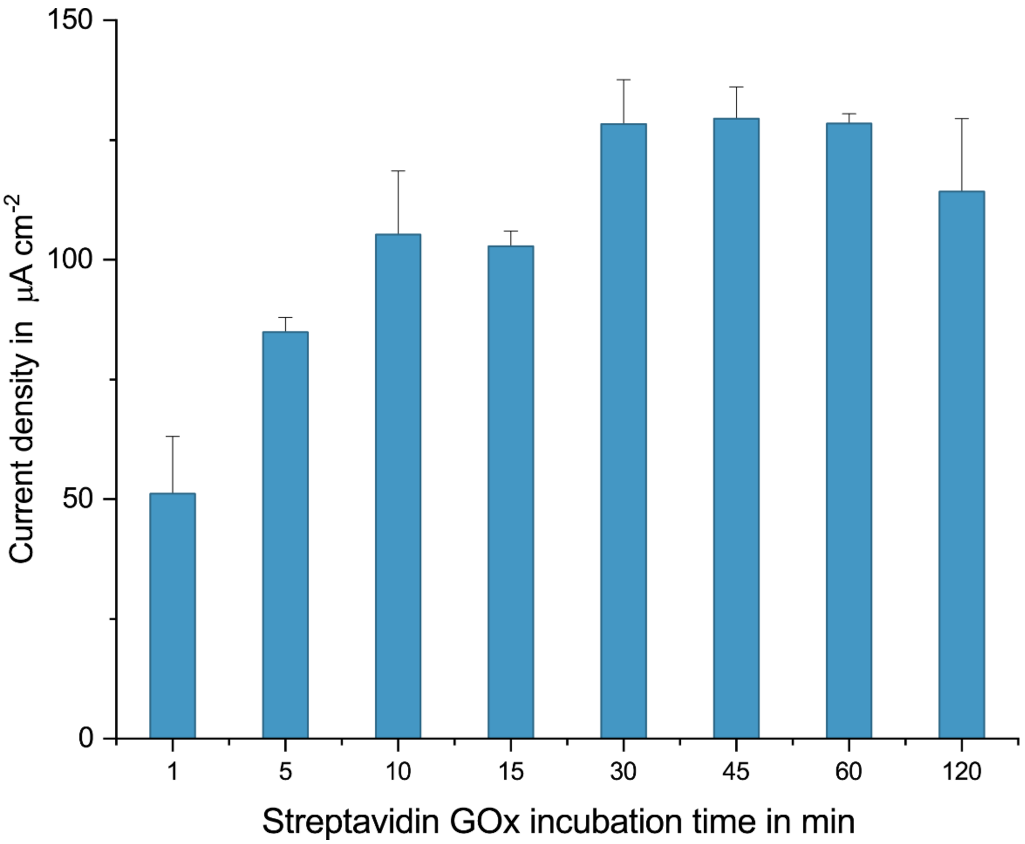

Figure S9: Comparison of different Streptavidin-GOx (10 µg ml^{-1}) incubation times. For ß-lactam measurements, unless otherwise stated, incubation time of Str-GOx was selected as 15 minutes. Error bars represent ± SD of n = 4 replicates.

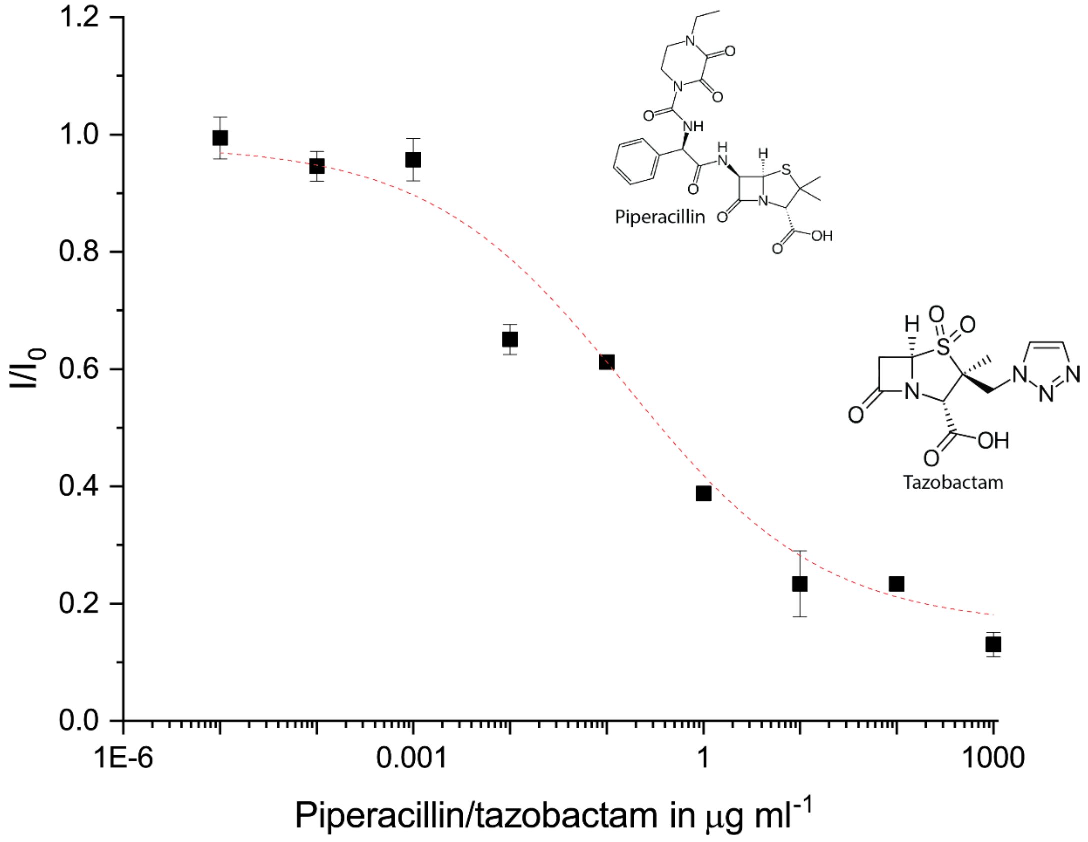

Figure S10: Calibration curve of the piperacillin/tazobactam in physiological PBS. The results were fitted with a 4-parametric logistic fit, resulting in a limit of detection of 0.056 µg ml^{-1}. Having high sensitivity, the optimized assay enables working with very diluted samples (up to 1,000 times) and detecting antibiotic concentration in these diluted samples. Accordingly, using only one calibration curve, drug concentrations in different samples can be calculated. All error bars represent ± SD of n = 4 replicates.

9. Measurement setup and process

To perform the amperometric signal readout, the microfluidic biosensor needs to be electrically and fluidically integrated into the measurement setup. For this purpose, 2 different PMMA-milled custom-made chip holders were fabricated for both single-analyte (milab) and multiplexed biosensors (Biosensor X).

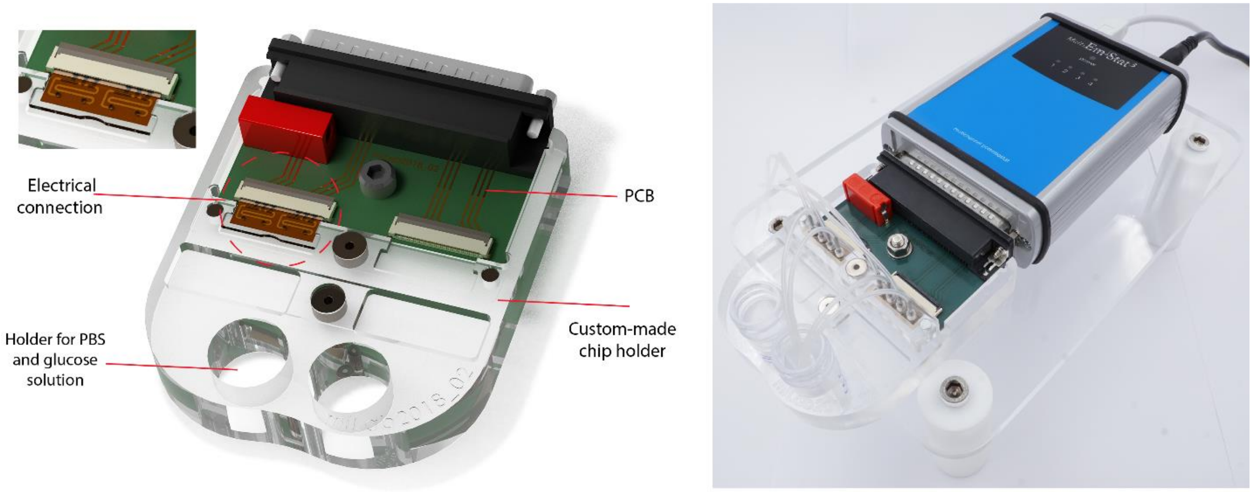

Figure S11: Rendered image of custom-made PMMA chip holder together with the single-analyte microfluidic chip and PCB (left) The measurement setup consists of a chip holder, the microfluidic chip, working solutions (PBS and glucose), potentiostat and a syringe pump. Working solutions are withdrawn via the syringe pump from the channel inlet to outlet of the microfluidic biosensor (right). Reproduced with permission[5].

The measurement procedure is same for both miLab and Biosensor X. A laptop is connected to the potentiostat that is controlled via the MultiTrace 4 software (PalmSens BV, The Netherlands), which visualizes the signals measured. For a constant fluid flow through the biosensor chip, the syringe pump PHD ULTRA™ 4400 (Harvard Apparatus, USA) is used in withdraw-mode, pulling the fluid from a reservoir through the microfluidic chip into the syringes. The flow rate is kept constant throughout the measurement, and set as 20 and 10 µl min^{-1} for miLab and Biosensor X, respectively. The pump also enables the programming of individual steps for withdrawing or infusing a fluid. By programming the pump, the stop-flow protocol (2- and 5-minute stop phases)

employed in this work can be applied and automated (Figure S12). Throughout the measurements, four 2.5-ml syringes (Hamilton Gastight, USA) for miLab and a 1-ml syringe (Hamilton Gastight, USA) for Biosensor X were used.

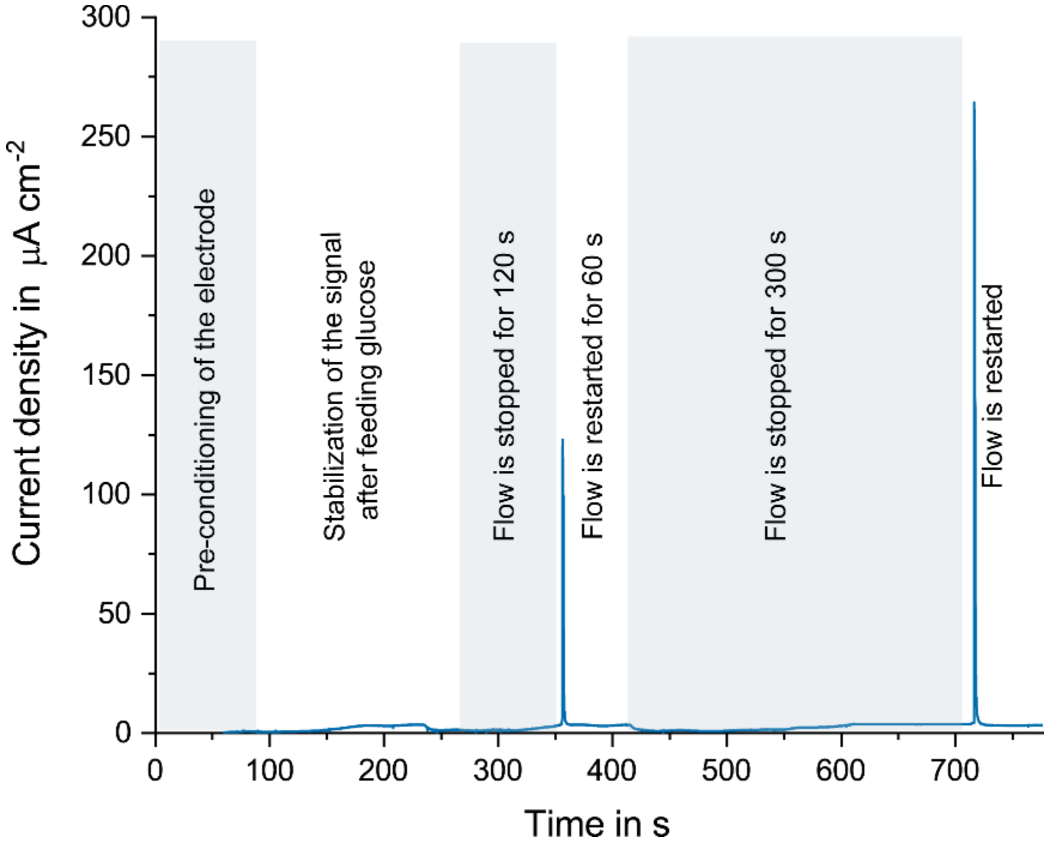

Figure S12: Readout procedure of the amperometric biosensor. After the preconditioning step of the working electrode (60 seconds), the 40 mM-glucose solution is introduced, and the signal stabilizes over the next couple of minutes. The flow of the glucose solution is stopped for 2 minutes, resulting in a first measurement peak and then restarted, followed by second stop for 5 minutes, resulting in the final measurement peak.

10. Sample pre-processing and dilution integrity

The first critical step was to determine the correct dilution factor for each sample type. Selection of the dilution factor as well as the dilution integrity for the plasma samples diluted at different rates were demonstrated. After optimization studies dilution factors were found to be: 1:500 for plasma, 1:750 for urine and 1:100 for saliva. For EBC measurements, since the expected concentration is already small, and dilution was performed to increase salt content as 9:10.

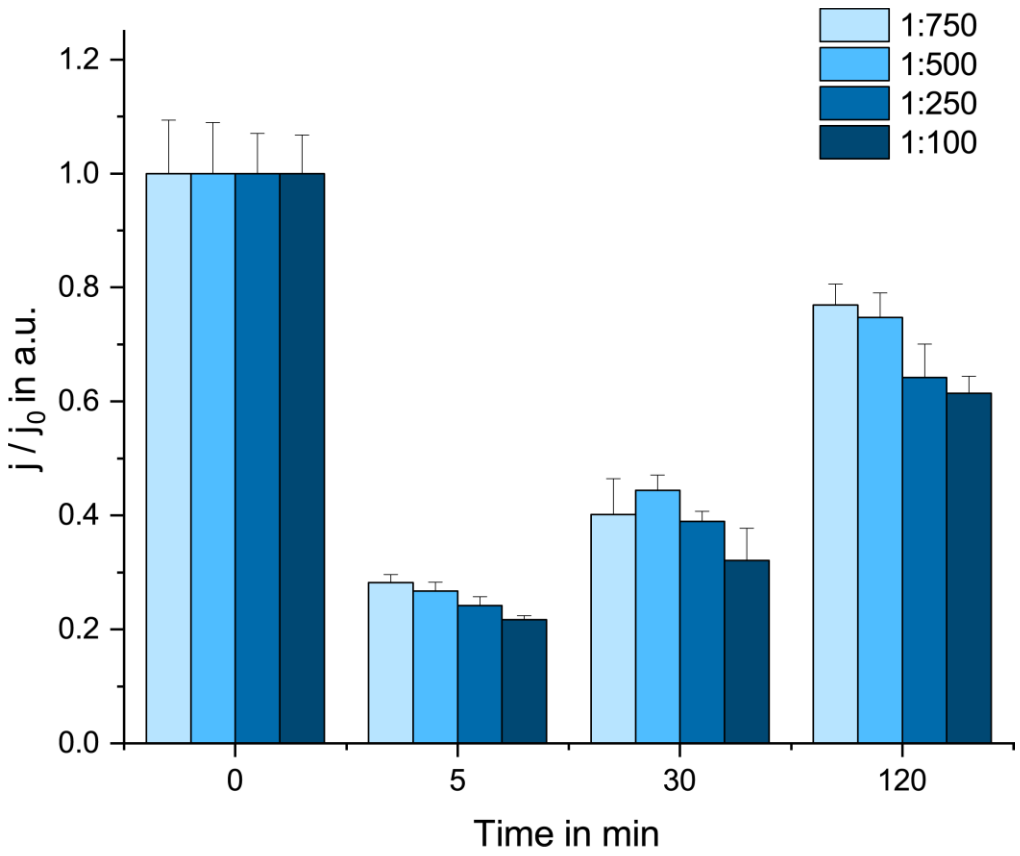

Figure S13: Demonstration of the dilution integrity for plasma samples taken from the animal given overdose antibiotics over a time period of 120 minutes. The same clearance effect was observed regardless of the dilution factor. For further measurements, dilution factor for the plasma samples of overdosed animals was selected as 1:500. Error bars represent ± SD of n = 4 replicates.

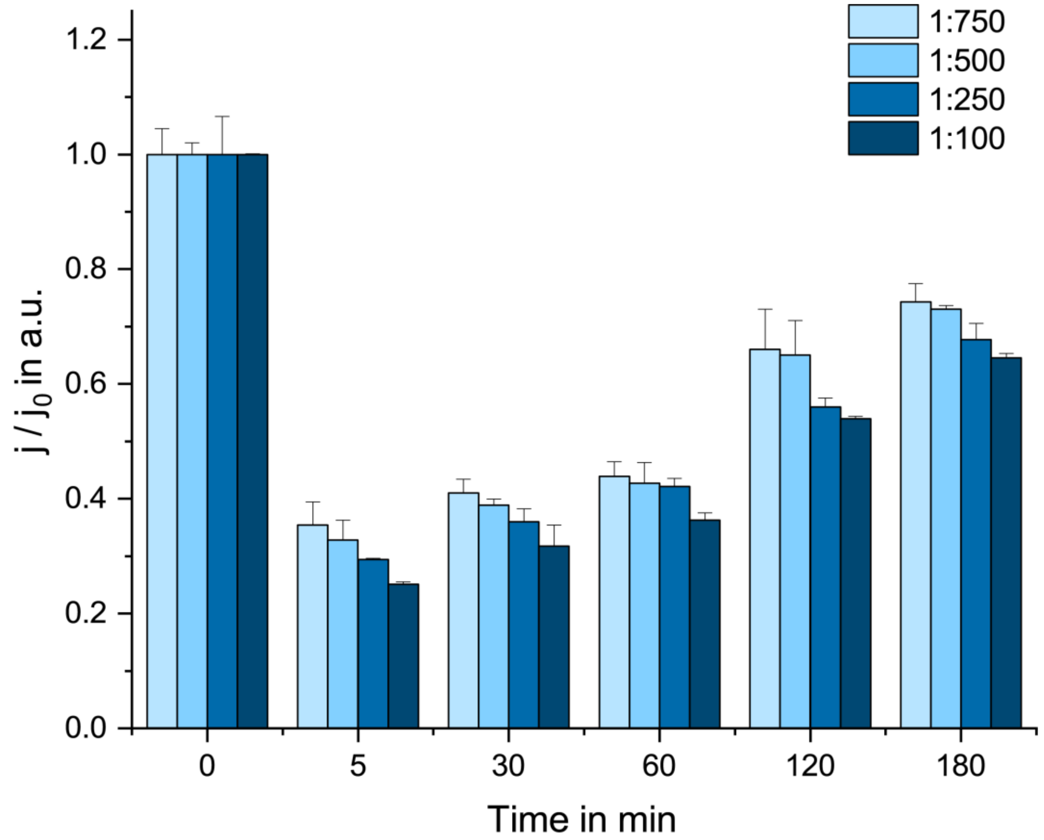

Figure S14: Study of the dilution integrity for plasma samples taken from the animal given normal-dose antibiotics over a time period of 180 minutes. The same clearance effect was observed regardless of the dilution factor. For further measurements, dilution factor for the plasma samples of normal-dosed animals was selected as 1:500. Error bars represent ± SD of n = 4 replicates.

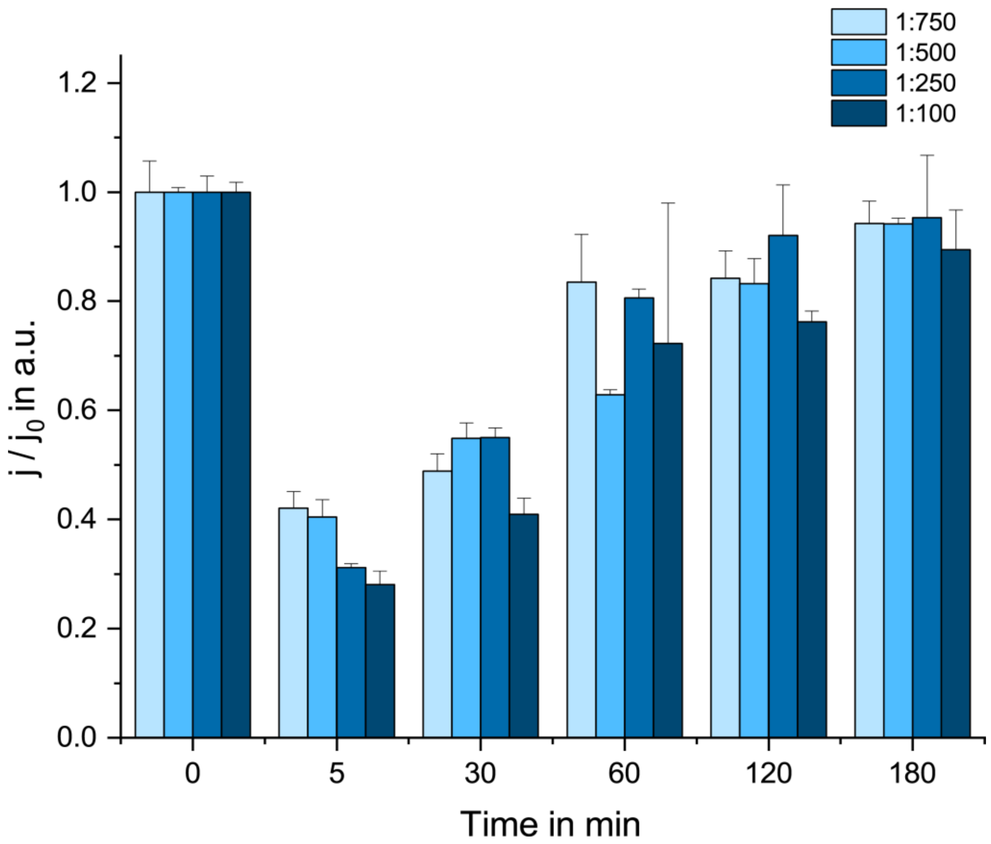

Figure S15: Comparison of the dilution integrity for plasma samples taken from the animal given underdose antibiotics over a time period of 180 minutes. The same clearance effect was observed regardless of the dilution factor. For further measurements, dilution factor for the plasma samples of underdosed animals was selected as 1:500. Error bars represent ± SD of n = 4 replicates.

11. Effect of an external drug on the measured signal

During the on-site animal experiments, drug concentrations followed the dosage regimen quantitatively. Nonetheless, during the concentration measurements, one anomaly was observed in the 120-minute-sample of the normal-dosed pig. In order to understand the source of this deviation, the animal's experiment log-book was checked, which revealed an emergency dosage of propofol (an anesthetic drug) to maintain anesthesia. This possibility was then confirmed with an *in vitro* test, where a similar concentration of propofol was spiked in a whole blood sample.

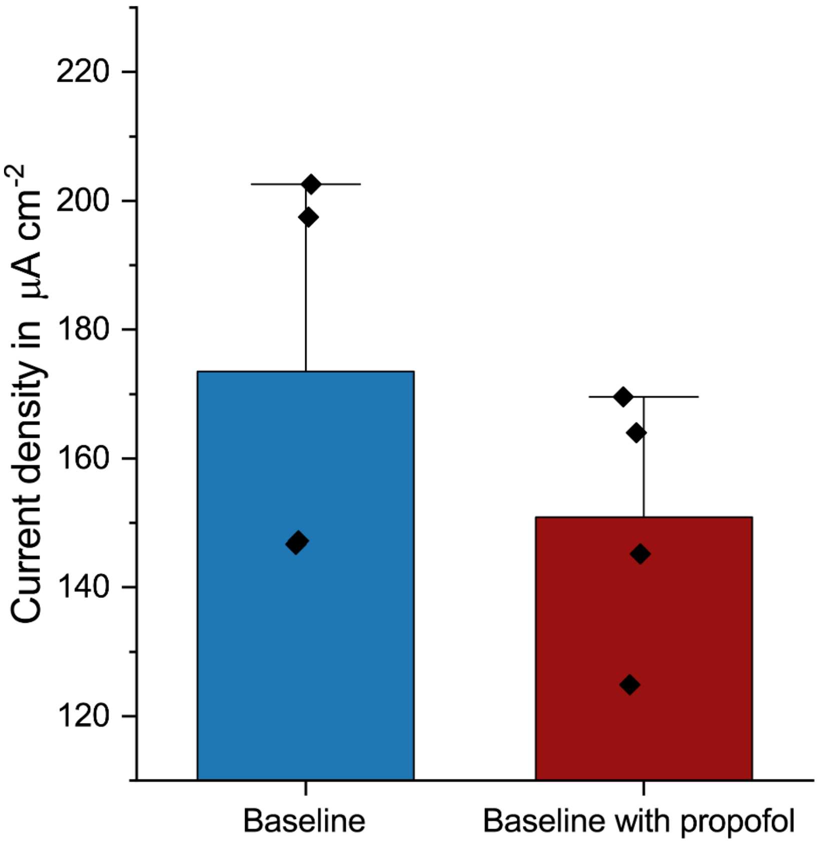

Figure S16: Demonstration of the external drug effects on the antibiotic measurement. Whole blood samples were taken from normal-dosed animal spiked with stimulated dosage of propofol. A short-term interference was caused by the presence of propofol. In the normal experimental setting, samples were taking from the animals following surgical intervention and a stabilization phase of 15 min, which eliminates the risk of interference of anaesthetics. Bar plot for n = 4 replicates. Error bars represent ± SD.

12. Gold standard validation

Benchmarking of our system was performed via comparing the plasma piperacillin concentrations measured with HPLC (gold standard) with the ones gauged with our biosensor. The plasma samples of pig given normal dose of antibiotics were collected before and 5-300 minutes after injection of the antibiotic, aliquoted and stored at –80 °C until measurement.

The details of gold standard measurement method are described in[6]. In addition to this method, plasma samples are deproteinized with the same volume of methanol. After a 10-minute centrifugation at 13,000 rpm, the samples are filtered through a PTFE membrane filter (0.2 µm) and then injected into HPLC. The deproteinisation improves significantly the life time of the NH_2 guard column (max. 50 samples without deproteinisation in comparison to more than 250 samples with deproteinisation). A Dionex Ultimate 3000 HPLC (Thermo Fisher Scientific, Germany) with column switching is utilized for analyzing plasma samples. The in-line extraction system consists of a Knauer Eurospher NH_2 guard column (2534 mm) and in-line filter (2-mm sieve), Merck (Darmstadt, Germany). A Macherey Nagel MN CC125/4 Nucleodur C18 PYRAMID 5µ is used as analytical column.

Standards to study linearity were made by dissolving piperacillin and tazobactam Kabi (4/0.5 g Lot: 18T3899) which leads an aqueous stock solution of piperacillin and tazobactam in a range of 1–16 mg ml^{-1}. Specificity of the system was tested with acyclovir, cefuroxime, ciprofloxacin, doxycycline, imipenem, meropenem, ofloxacin, oxycodone, penicillin V, sulfamethoxazole, trimethoprim and vancomycin. Linearity was shown in a range from 16 to 800 µg ml^{-1} for piperacillin ($r = 0.9996$) and from 2 to 100 µg ml^{-1} for tazobactam ($r = 0.9997$). Calibration was performed by measuring 7 ß-lactam concentrations and $n = 6$ for each measurement point with an accuracy less than 10%. Quantitation limit was estimated as 1 µg ml^{-1} for piperacillin and 2 µg ml^{-1} for tazobactam.

To convert obtained current densities to the drug concentrations, on-chip calibration curve (Figure S10) was used. Since the working principle of our biosensor depends on the measurement of drugs with ß-lactam ring (which is the case for both piperacillin and tazobactam), it is not possible to identify piperacillin and tazobactam levels individually. Instead, our platform measures the total amount of free piperacillin/tazobactam in the sample. HPLC measurements, on the other hand, reports free piperacillin and tazobactam plasma levels separately. Consequently, we summed up the reported gold standard piperacillin and tazobactam levels and compared this total amount

with the one gauged by our sensor (n = 7). However, only tazobactam levels of 5, 30 and 60 minutes could be analyzed and reported via gold standard method; as the tazobactam levels in the sample were below the limit of quantification at other time points.

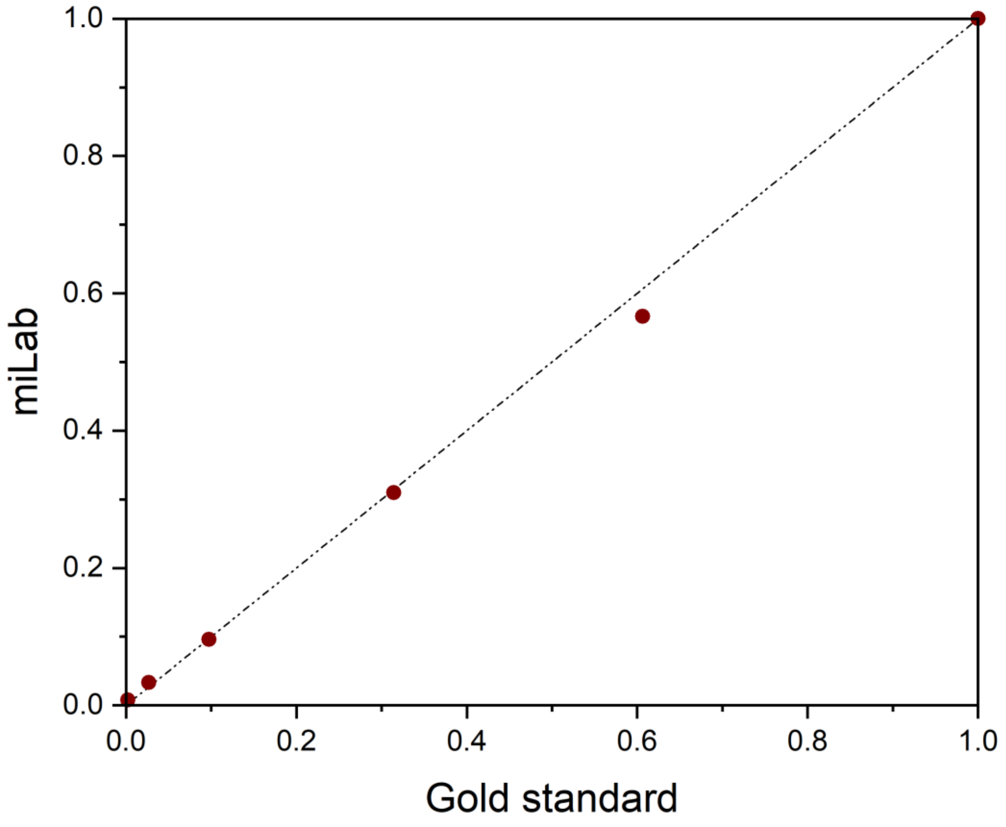

Figure S17: Scatter plot of drug concentrations measured with gold standard and miLab. Almost 1:1 relationship is observed at each data points.

13. Validation of Biosensor X with milab

Validation of Biosensor X was performed via comparing current densities obtained by the analysis of piperacillin/tazobactam in plasma samples. The signal intensities measured are flow rate-dependent and thus, there is a difference between the current densities of miLab and Biosensor X measurements. However, the normalized values shown in Figure 5 (in the manuscript) are in a good agreement.

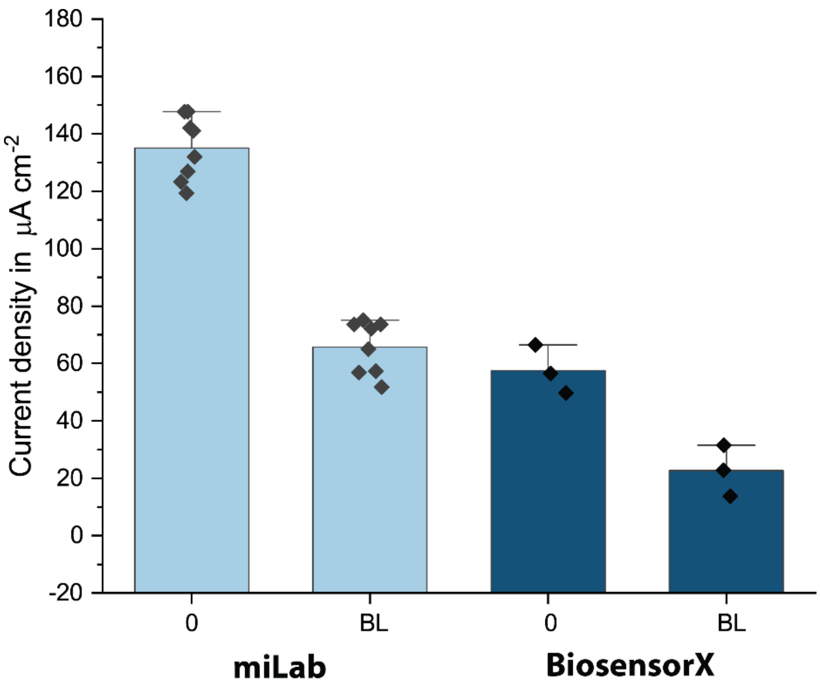

Figure S18: Comparison of measured current densities with miLab and Biosensor X for the plasma samples of animal given normal dosage of piperacillin/tazobactom. To compare the clearance behavior of the drug both 0 (before antibiotic administration) and BL (5-minute after antibiotic administration) are analyzed (n=7 and n=3 for miLab and Biosensor X, respectively).

Table S3: Comparison of our platform with available methods utilized for ß-lactam quantification[7–13].

Method	Sensitivity	Specificity	Sample volume	Pre-processing	Sample-to-result
Chromatography[6-10]	0.1-10 µg ml^{-1}	Assay-free	Low	High	High
ELISA[11-12]	0.5-2 µg ml^{-1}	Requires a specific antibody for each ß-lactam	High	Middle	Low
miLab (this work)	56 ng ml^{-1}	Antibody-free assay for all ß-lactams	Low	Low	Low

References

[1] R. Bruch, A. Kling, G. A. Urban, C. Dincer, *J. Vis. Exp.* **2017**, 1.

[2] R. Bruch, J. Baaske, C. Chatelle, M. Meirich, S. Madlener, W. Weber, C. Dincer, G. A. Urban, *Adv. Mater.* **2019**, DOI 10.1002/adma.201905311.

[3] R. Bruch, C. Chatelle, A. Kling, B. Rebmann, S. Wirth, S. Schumann, W. Weber, C. Dincer, G. Urban, *Sci. Rep.* **2017**, *7*, 1.

[4] T. H. J. Niedermeyer, M. Strohalm, *PLoS One* **2012**, DOI 10.1371/journal.pone.0044913.

[5] R. Bruch, Multiplexed MiRNA Biosensor for Pediatric Cancer Diagnostics, University of Freiburg, **2020**.

[6] R. Trittler, M. Ehrlich, T. J. Galla, R. E. Horch, K. Kümmerer, *J. Chromatogr. B* **2002**, *775*, 127.

[7] S. Van Vooren, A. G. Verstraete, *Biomed. Chromatogr.* **2021**, *35*, e5092.

[8] L. A. Decosterd, T. Mercier, B. Ternon, S. Cruchon, N. Guignard, S. Lahrichi, B. Pesse, B. Rochat, R. Burger, F. Lamoth, J. L. Pagani, P. Eggimann, C. Csajka, E. Choong, T. Buclin, N. Widmer, P. André, O. Marchetti, *J. Chromatogr. B* **2020**, *1157*, 122160.

[9] J. D'Cunha, Ronilda Bacha, Thanh Younga, Beth Ann Lib, Peizhi Nalbanta, Demet Zhangc, G. Winokurd, Patricia An, *Antimicrob. Agents Chemother.* **2018**, DOI 10.1128/AAC.00861-18.

[10] S. FB, R. MS, R. JA, R. TA, *J. Chromatogr. B. Analyt. Technol. Biomed. Life Sci.* **2014**, *960*, 134.

[11] S. Cairoli, R. Simeoli, M. Tarchi, M. Dionisi, A. Vitale, L. Perioli, C. Dionisi-Vici, B. M. Goffredo, *Biomed. Chromatogr.* **2020**, *34*, e4880.

[12] D. Fage, G. Deprez, B. Fontaine, F. Wolff, F. Cotton, *Talanta* **2021**, *221*, 121641.

[13] J. Adrian, D. G. Pinacho, B. Granier, J.-M. Diserens, F. Sánchez-Baeza, M.-P. Marco, *Anal. Bioanal. Chem. 2008 3915* **2008**, *391*, 1703.
